# The Effects of Artificially Dosed Adult Rumen Contents on Abomasum Transcriptome and Associated Microbial Community Structure in Calves

**DOI:** 10.3390/genes12030424

**Published:** 2021-03-16

**Authors:** Naren Gaowa, Wenli Li, Brianna Murphy, Madison S. Cox

**Affiliations:** 1 State Key Laboratory of Animal Nutrition, Beijing Engineering Technology, Research Center of Raw Milk Quality and Safety Control, College of Animal Science and Technology, China Agricultural University, No.2 Yuanmingyuan West Road, Haidian, Beijing 100193, China; narengaowa@cau.edu.cn; 2 The Cell Wall Utilization and Biology Laboratory, USDA Agricultural Research Service, US Dairy Forage Research Center, Madison, WI 53706, USA; bmurphy8@wisc.edu; 3 Department of Bacteriology, University of Wisconsin-Madison, Madison, WI 53706, USA; mcox4@wisc.edu; 4 Microbiology Doctoral Training Program, University of Wisconsin-Madison, Madison, WI 53706, USA

**Keywords:** artificial dosing, abomasum transcriptome, young calves

## Abstract

This study aimed to investigate the changes in abomasum transcriptome and the associated microbial community structure in young calves with artificially dosed, adult rumen contents. Eight young bull calves were randomly dosed with freshly extracted rumen contents from an adult cow (high efficiency (HE), *n* = 4), or sterilized rumen content (Con, *n* = 4). The dosing was administered within 3 days of birth, then at 2, 4, and 6 weeks following the initial dosing. Abomasum tissues were collected immediately after sacrifice at 8 weeks of age. Five genera (*Tannerella, Desulfovibrio, Deinococcus, Leptotrichia, and Eubacterium*; *p* < 0.05) showed significant difference in abundance between the treatments. A total of 975 differentially expressed genes were identified (*p* < 0.05, fold-change > 1.5, mean read-counts > 5). Pathway analysis indicated that up-regulated genes were involved in immune system process and defense response to virus, while the down-regulated genes involved in ion transport, ATP biosynthetic process, and mitochondrial electron transport. Positive correlation (r > 0.7, *p* < 0.05) was observed between *TRPM4* gene and *Desulfovibrio*, which was significantly higher in the HE group. *TRPM4* had a reported role in the immune system process. In conclusion, the dosing of adult rumen contents to calves can alter not only the composition of active microorganisms in the abomasum but also the molecular mechanisms in the abomasum tissue, including reduced protease secretion and decreased hydrochloric acid secretion.

## 1. Introduction

The ruminant stomach has four functional compartments: rumen, reticulum, omasum, and abomasum for the efficient use of plant materials. The abomasum is the last stomach compartment of the ruminant stomach. It is a secretory stomach, anatomically and functionally similar to the stomach of non-ruminants. Because it is a connection between the pre-intestinal and digestive tract in ruminants, the abomasum plays a major role in the acid hydrolysis of microbial and dietary proteins, facilitating further digestion and absorption of proteins in the small intestine. So far, the majority of work on the ruminant stomach has mainly focused on the rumen, largely due to its relatively easy access. Despite the important role of abomasum in nutrient digestion, we currently have very limited knowledge about its physiology and underlying molecular mechanisms under different feeding treatments. Much more work is needed to advance the full understanding of the digestive function of abomasum and its contribution to the health and efficiency of gastrointestinal tract (GIT) in ruminants.

During postnatal development, ruminants go through a suite of complex morphological changes, which allow them to efficiently digest milk as its primary diet in the neonatal phase and then to transition to a plant-based diet in the post-weaning phase [1]. From birth to the first three weeks, the primary colostrum or mature milk substitute is the liquid feed administered directly into the abomasum via the esophageal groove [2]. At this stage, colostrum contains the important source for GIT bacterial colonization, accounting for the initial colonization of >200 species [3]. Significant anatomical development of the rumen, including rumen volume and growth of the rumen papilla, co-occurred with increased solid feed intake [2,4,5]. Additionally, further colonization of a microbial community will continue and qualitative changes in nutrient digestion and metabolism pathways have been reported [6]. At this stage, abomasum has transitioned from digestion of colostrum to digestion of the microbial protein and solid feed protein, associated with the colonization of its own microbial community [7]. However, the early development and the microbial colonization of the abomasum in young calves remain largely unexplored.

Since the emergence of RNA-seq as an approach for the study of physiological changes in human and model organisms [8], application of transcriptome analysis in the stomach of ruminant has been expanding [9]. On the one hand, researchers focused on obtaining a more complete understanding of the molecules and pathways in rumen papillae between low and high feed efficiency in adult beef cattle [10]. On the other hand, the effects of dietary physical [11] or nutritional factors [12] on transcriptome changes of rumen papillae have been evaluated. Of note, RNA-seq based studies in the abomasum of ruminants are limited. Few studies evaluated the effects of different feeding strategies on the structure and function of the abomasum in cattle. For example, previous studies demonstrated that ruminal butyrate [9] and the increased proportion of dietary concentrate [13] may affect not only the rumen but also other parts of the GIT, such as the omasum, abomasum, and proximal small intestine. The microbiota within the four stomachs in ruminant could pass from one segment to another with the digest [7]. One intriguing specialization of abomasum involves its ability to handle large masses of bacteria [14]. In contrast to the stomach of non-ruminants, the abomasum secretes lysozyme, which efficiently breaks down bacterial cell walls [14]. According to Lei’s study, *Lactobacillus* is one of the beneficial bacteria that lowers the pH in the abomasum, which allows the abomasum to act as a barrier, preventing the transfer of microorganisms to the hindgut in young ruminants [7].

Artificial dosing of rumen content using the cud from a healthy cow to treat a sick recipient animal was practiced long before our understanding of the important roles of rumen microorganisms [15]. Ruminal microbes have been used to treat indigestion [16], detoxify the toxic compounds in the feed [17], and enhance the surgical repair of left-displaced abomasum [18]. Furthermore, the potential medicinal role of a healthy microbial community in GIT is also tested in humans, especially the application of fecal microbiota transplantation to treat digestive disorders in humans [19]. Many studies have shown that initial microbial colonization occurs immediately after birth and that key bacterial phyla and genera are colonized heavily in GIT during the first few days of life [6,20,21]. Numerous human and mouse studies have reported the importance of early establishment of gut microbiota on host health, energy intake and storage [22]. In our previous study, in the young calves artificially dosed with rumen content from adult cow, we observed that the dosing can not only affect the rumen epithelium microbial community, but also the host liver transcriptome in young calves [23], indicating the potentially profound impact of early dosing of adult rumen content on the GIT tract in young calves. The abomasum is the ‘true stomach’ in ruminants and known for its vital role in hydrochloric acid secretion, protein digestion, and nutrient transfer. The purpose of this study is to investigate the effects of early dosing in the microbial colonization in abomasum and its associated host transcriptome changes in the abomasum (especially the genes related to hydrochloric acid secretion and encoding digestive enzymes). Our results provided a snapshot into the microbiota changes in the abomasum. Collectively with our previously published work in other GIT tissues, the knowledge gained in this study will contribute to the understanding of GIT homeostasis maintenance and intervention with feeding manipulation in calves during early development.

## 2. Materials and Methods

### 2.1. Experimental Design and Sample Collection

Throughout the experiment, all animals involved in this study were fed and watered according to the herd standard practices at the USDA Dairy Forage Research Center farm. All animal protocols were approved by University of Wisconsin-Madison, Institutional Animal Care and Use committee (protocol number: A01427). Animal care and use, and all methods involved in this study were performed in accordance with the relevant guidelines and regulations by the Animal Welfare Act from US Department of Agriculture and by the Federation of Animal Science Societies.

This study was part of a larger experiment, which can separate into two parts. Firstly, milk production efficiency index (as calculated by energy-corrected milk divided by dry matter intake (ECM/DMI)) was used to select the high efficiency (HE) and low efficiency (LE) adult cows as described by Jewell et al. [24]. One adult HE cow (4262; age = 5 years, ECM/DMI = 1.8; The diet composition of this donor cow was described in detail in Jewell et al. [24]) was used as the donor of rumen content in this study. Secondly, 8 young bull calves were randomly assigned into two groups: (1) control group (Con; supplemented with autoclaved rumen content, *n* = 4) and (2) treatment group (HE; supplemented with inocula from the HE donor cow, *n* = 4). As mentioned in the paper of Li et al. [23], the control inocula were prepared by autoclaving the combined inocula (50% from each) from the HE donor cow and the LE donor cow. Experiment was carried out from birth to 56 days. The inocula was administered within 3 days after birth, then at 2, 4, and 6 weeks following the initial dosing [23]. Until 8 weeks of age, the 4 calves in each group were sacrificed and the abomasum tissues were collected immediately after euthanization. Upon collection, tissues were rinsed in PBS to remove any feed particles, and cut with sterilized scalpels into small fragments (4–5 mm^2^). Tissues were put into Eppendorf safe-lock tubes (Eppendorf North America, Hauppauge, NY, USA), flash frozen in liquid nitrogen and stored at −80 °C for long-term storage.

### 2.2. RNA Extraction, Quantification and Whole Transcriptome Sequencing

This section followed the protocols described in our publications [23]. For RNA extraction, 30–50 mg raw tissue was grinded using Precellys lysing kit (Bertin Technologies SAS, Montigny-le-Bretonneux, France). The RNAs were extracted using miRNeasy kit (Qiagen, Germantown, MD, USA) following manufacturer’s instructions. Quality of extracted RNAs was appraised using a Bioanalyzer RNA 6000 nano kit (Agilent Technologies, Santa Clara, CA, USA). The RNA samples with RNA integrity number ≥ 7.8 were pursued by RNA quantification assessment using Qubit (Thermo Fisher Scientific, Waltham, MA, USA). RNA-sequencing library preparation was done using TruSeq Stranded Total RNA Library Prep Kit with Ribo-Zero Gold (Illumina, San Diego, CA, USA) following manufacturer’s protocol. One μg of total RNA in each sample was used for sequencing library preparation. Quantification of prepared cDNA library was performed using Kapa quantification kit (Kapa systems) using ABI7300 instrument (Thermo Fisher Scientific). The library products were sequenced via an Illumina NextSeq 500 sequencer.

### 2.3. Bioinformatics and Data Analysis

The quality of sequencing raw reads was checked using FASTQC (Version 0.11.8, https://www.bioinformatics.babraham.ac.uk/projects/fastqc/, accessed on 10 February 2021). High-quality sequencing reads were aligned to the cattle reference genome (Bos taurus UMD 3.1) using HISAT2 [25]. Raw read-counts for each gene were obtained using HTSeq (v.0.6) [26]. Analysis of differentially expressed genes (DEGs) was performed using the bioinformatics tool DESeq2 [27] in R with raw read-counts calculated by HTSeq as the input. Read-count normalization was performed using the regularized logarithm (rlog) method provided in DESeq2 with *p*-values corrected for multiple testing using the Benjamini-Hochberg method [28]. Genes with less than five normalized read-counts were excluded from further analysis. DEGs were determined by *p*-value (cutoff of 0.05) and the fold-change (cutoff of 1.5) by DESeq2. Gene function annotation and pathway analysis were performed using the Database for Annotation, Visualization, and Integrated Discovery (DAVID v.6.8, https://david.ncifcrf.gov/, accessed on 10 February 2021).

To obtain rRNA reads from the abomasum microbial community, total RNA-seq raw reads were first mapped against the cattle genome reference (NCBI, UMD 3.1) using STAR [29]. Unmapped reads, which are non-cattle reads, were mapped to rRNA reference databases provided by SortMeRNA [30] to obtain microbial rRNA reads. Microbial rRNA reads were used for downstream microbial genus-level classification using Kraken [31]. To compare the microbial community expression differences between high efficiency (HE) and sterilized rumen content (Con) groups, Kruskal–Wallis non-parametric test was employed using the normalized read-counts from Kraken [31], which was used as the measurement of microbial abundance. The normalized read-count was calculated using the method described in Li et al. [32]. Briefly, the total mapped reads at each genus was divided by the “per million factor”, which is the combined number of reads mapped to all genera divided by 1,000,000. The correlation between gene expression and microbial genus-level abundance was done with Pearson correlation analyses using the “cor” function in RStudio, the statistical computing and graphics software (version 3.4.0), with default parameters. DEGs involved in immune response and the bacterial genera with significantly different abundance in abomasum were included in the correlation analysis. The “corrplot” package was used for plotting.

### 2.4. RT-qPCR Verification of Target Genes

Four randomly selected genes were analyzed using reverse transcriptase quantitative PCR (RT-qPCR) to assess their differential expression between Con and HE groups. These genes were: lysozyme C-2 (*LYZ2*), pepsinogen 5 group I (pepsinogen A) (*PGA5*), tetraspanin 3 (*TSPAN3*), and potassium two pore domain channel subfamily K member 5 (*KCNK5*). *LYZ2* encodes lysozyme C-2, which can digest symbiotic bacteria [33]. The ruminant gastric lysozymes are much more resistant to inactivation by pepsin than other lysozymes [34]. *PGA5* encodes a protein precursor of the digestive enzyme pepsin, a member of the peptidase A1 family of endopeptidases. The protein encoded by *TSPAN3* gene is a member of the transmembrane 4 superfamily, also known as the tetraspanin family [35]. Gene *KCNK5* encodes one member of the superfamily of potassium channel proteins, which is highly sensitive to external pH and may play an important role in potassium transport [36].

cDNA synthesis was done using High Capacity cDNA master mix (Life Technologies, Carlsbad, CA, USA) with 2000 ng of total RNA. Gene-specific, Taqman assay probes were ordered from Thermo Fisher (Thermo Fisher Scientific). All qPCR reactions were performed using the QuantStudio 5 fast system (Thermo Fisher Scientific). The PCR conditions were set as follows: one step of uracil-N-glycosylase (UNG) [37] treatment at 50 °C for 2 min, followed by an initial denaturation/activation step at 95 °C for 10 min, then 40 cycles at 95 °C for 15 s and 60 °C for 60 s. The fold change in gene expression was obtained following normalization to two reference genes, beta-actin (*ACTB*) [38] and hydroxymethylbilane synthase (*HMBS*) [39]. These two reference genes were not among the DEGs between HE and Con groups. Both of them were used as an endogenous reference gene for all reactions. Final RT-qPCR data were obtained by 2^−ΔΔCt^ method [40].

## 3. Results

### 3.1. RNA Quality, Sequencing Reads and Total Number of Expressed Genes

**Figure 1:**
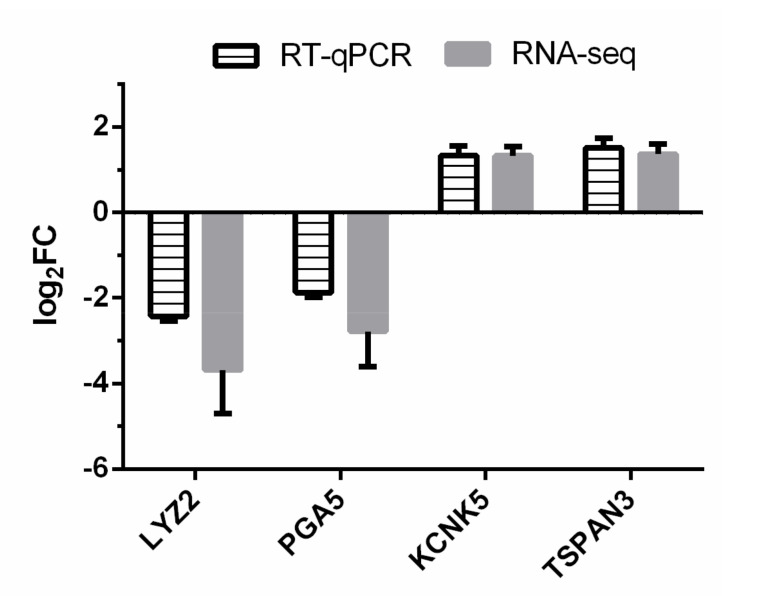
RT-qPCR confirmation of four differentially expressed genes (DEGs) identified by RNA-seq. Log_2_FC (high efficiency (HE) vs. sterilized rumen content (Con)) of target genes were calculated for both RNA-seq and RT-qPCR methods.

On average, 10,111,666 ± 1,534,352 (mean ± s.e.) sequence reads for all the samples from both Con and HE groups were obtained. After sequence alignment, 77.6 to 77.8% of the reads from each sample was mapped to the cattle reference genome. The expression profile of the selected genes was successfully confirmed by RT-qPCR method (Figure 1).

### 3.2. Differentially Expressed Genes 

**Figure 2:**
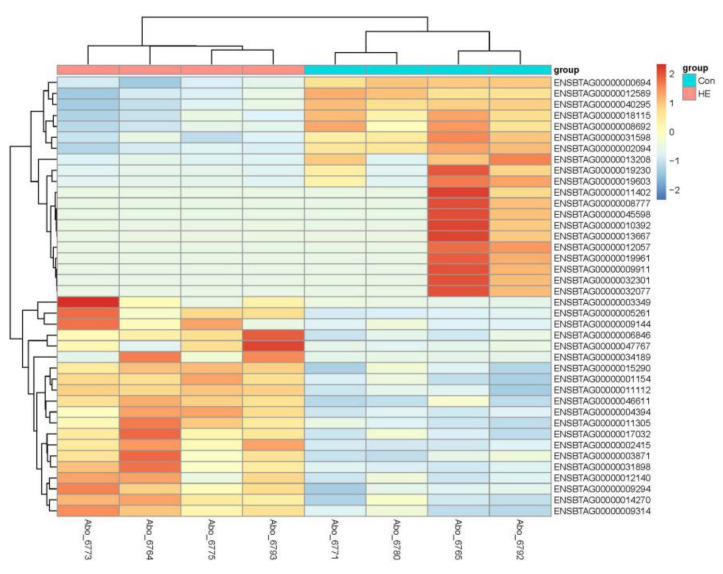
Heatmap showing top 40 DEGs between treatments. The log2 ratio values of DEG abundance were used for cluster analysis with the R pheatmap package. Red and blue indicate relative over- or under-expression of genes, respectively.

There were 975 genes differentially expressed between the Con and HE groups (*p* < 0.05 and fold-change > 1.5) with 348 up-regulated genes (URGs) and 627 down-regulated genes (DRGs) in the HE group in comparison to the Con group (Table S1). The top 40, most significant DEGs clearly separated the two treatment groups (Figure 2).

### 3.3. Functional Annotation of Differentially Expressed Genes

**Figure 3:**
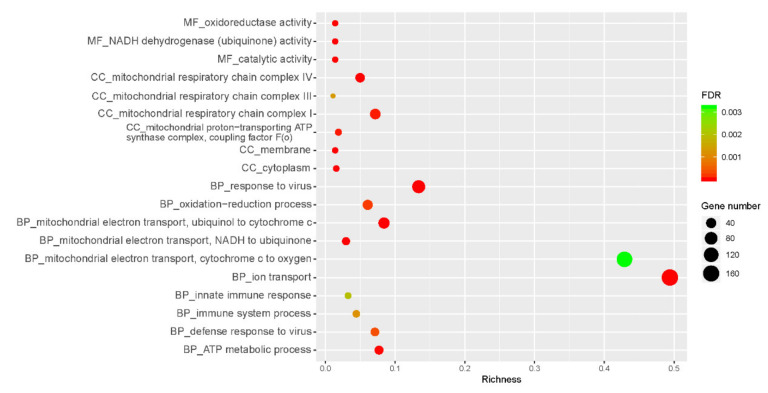
Function annotation-GO analysis of DEGs. FDR: false discovery rate; MF: molecular functions; BP: biological process; CC: cellular component. The x-axis is the richness calculated from the numbers of DEGs in a term divided by all the genes included in that term. As shown on the right side of the figure, the size of the circumference correlates with the number of genes.

As shown in Figure 3, Gene Ontology (GO) analysis indicated that DRGs were enriched in the cellular component in the mitochondrial membrane, such as mitochondrial respiratory chain complex I, III, IV (GO:0005747, 32 genes; GO:0005750, 9 genes; GO:0005751, 9 genes; FDR < 0.05), and proton-transporting ATP synthase complex (GO:0000276, 9 genes, FDR < 0.05). For biological process, DRGs were predominantly enriched in the following GO terms: oxidation-reduction process (GO:0055114, 86 genes, FDR < 0.05), ion transport (GO:0006811, 46 genes, FDR < 0.05), ATP metabolic process (GO:0046034, 12 genes, FDR < 0.05), mitochondrial electron transport including ubiquinol to cytochrome c (GO:0006122, 10 genes, FDR < 0.05), NADH to ubiquinone (GO:0006120, 9 genes, FDR < 0.05) and cytochrome c to oxygen (GO:0006123, 7 genes, FDR < 0.05). Moreover, molecular functions enriched by DRGs included oxidoreductase activity (GO:0016491, 54 genes, FDR < 0.05), catalytic activity (GO:0003824, 39 genes, FDR < 0.05), and NADH dehydrogenase (ubiquinone) activity (GO:0008137, 19 genes, FDR < 0.05). For URGs in the HE group, they were enriched in the following GO terms: immune system process (GO:BP, GO:0002376, 26 genes, FDR < 0.05), innate immune response (GO:BP, GO:0045087, 24 genes, FDR < 0.05), defense response to virus (GO:BP, GO:0051607, 15 genes, FDR < 0.05), response to virus (GO:BP, GO:0009615, 11 genes, FDR < 0.05), membrane (GO:CC, GO:0016020, 167 genes, FDR < 0.05), and cytoplasm (GO:CC, GO:0005737, 145 genes, FDR < 0.05) (details shown in Table S2).

### 3.4. rRNA Transcriptome Analysis of Abomasum Microbial Community and Its Association with Host mRNA Expression Changes

**Figure 4:**
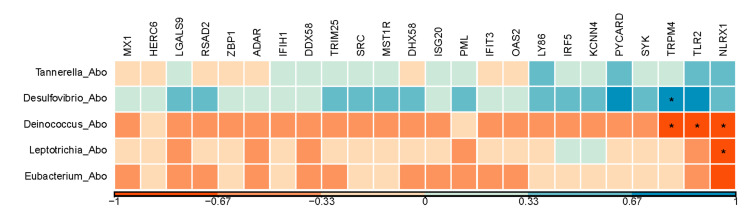
A Pearson correlation matrix between significantly changed active bacteria and the genes enriched in immune response. The scale of the colors is denoted as follows: the more positive the correlation (closer to 1), the darker the shade of blue; the more negative the correlation (closer to −1), the darker the shade of red. *: *p* < 0.05; Abo: abomasum tissue.

After using Kraken to classify the microbial genus-level, a total of 102 genera had been found in the abomasum (Table S3). Five of them showed significant difference in abundance between the treatments, with *Tannerella* and *Desulfovibrio* higher in abundance and *Deinococcus, Leptotrichia*, and *Eubacterium* lower in abundance in the HE group (Table 1, *p* < 0.05). *Desulfovibrio* was positively related to *TRPM4* gene, which has a reported role in immune system process [41] (Figure 4, r = 0.8, *p* < 0.05). Genera of *Deinococcus* were negatively related 3 URGs, involved in immune system process (Figure 4, *TRPM4, TLR2, NLRX1*; r = −0.8, −0.8, and −0.7, respectively; *p* < 0.05). Moreover, *Leptotrichia* were negatively related to *NLRX1* (Figure 4, r = −0.7, *p* < 0.05). More exact correlation coefficients and *p*-values between genes and genera were shown in Table S4.

## 4. Discussion

**Figure 5:**
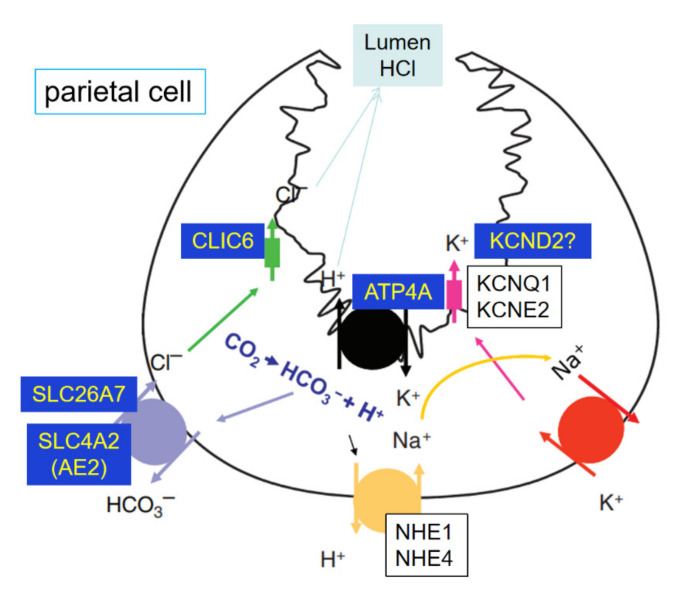
Ion transporters involved in hydrochloric acid (HCl) output of the parietal cell. *ATP4A, KCND2, SLC26A7, SLC4A2* and *CLIC6* were the DEGs in abomasum.

Ion transporters involved in hydrochloric acid (HCl) output of abomasum changed significantly. GO term annotation for the DRGs with the most significant fold-change between treated and control groups showed that ion transportation (GO:0006811; 46 genes, FDR < 0.05) is the most significant biological process identified in this study. For the top 10 most significant DRGs, there were 4 genes (*ATP4A, KCND2, SLC26A7*, and *CLIC6*) involved in the ion transportation process. Two of them (*KCND2* and *SLC26A7*) were also enriched in transmembrane transport (GO:0055085, 24 genes, *p* = 0.0013). As shown in Figure 5, 4 DRGs in our study were related to the hydrochloric acid (HCl) output in stomach parietal cells. ATPase H+/K+ transporting alpha subunit, *ATP4A* (Table S1 log_2_FC = −8.45, *p* < 0.001), codes the H^+^/K^+^ ATPase. The H^+^/K^+^ ATPase in the stomach is located in the stimulated state of the canaliculus and secretes gastric acid through electrically neutral ATP-dependent hydrogen-potassium exchange [42]. It could activate the digestive enzyme pepsin [43]. Many researchers have shown that the H^+^/K^+^ ATPase, which is controlled by the expression of *ATP4A,* is essential for acid secretion [44,45,46]. Although the pH of abomasum content was not measured in this study, lowered *ATP4A* expression suggested that calves in the HE group may have lower gastric acid secretion in abomasum. Solute carrier family 26 member 7, *SLC26A7* (Table S1 log_2_FC = −6.07, *p* < 0.001), solute carrier family 4 member 2, *SCL4A2* (log_2_FC = -2.39, *p* = 0.003) and channel chloride intracellular channel 6, *CLIC6* (log_2_FC = −3.69, *p* < 0.001) were related to the Cl^−^ export in parietal cells. *SLC26A7* is a vital Cl^−^/HCO_3_^−^ exchanger in the Cl^−^ import and HCO_3_^−^ extrusion pathway during acid secretion [47]. Moreover, *SLC26A7* functions as a pH_i_-regulated Cl^−^ channel with minimal HCO_3_^−^ permeability [48]. *SCL4A2* has a similar function as *SLC26A7* [49]. When *SCL4A2* is disrupted in mice, acid secretion disappeared, and the development of parietal cells was severely impaired [50]. Thus, it plays an important role in acid secretion and the development of the parietal cells. *CLIC6* encodes a member of the chloride intracellular channel family of proteins and associate with regions containing the H, K ATPase [42]. Potassium access from the lumen depends on the activation of potassium and chloride conduction via a KCNQ1/KCNE2 complex and CLIC6 [43]. In this study, *KCNQ1* and *KCNE2* were not down-regulated. However, potassium voltage-gated channel subfamily D member 2, *KCND2*, showed more than 64-fold decrease in expression in our experiment (Table S1 log_2_FC = −6.96, *p* < 0.001). This gene may be a key activation of the K and Cl conductance in abomasum. Acid secretion and its relation to flow of food material was documented in sheep [51,52]. In adult cattle, the abomasum has a lining folded into ridges, which produce gastric juices containing hydrochloric acids. Such an acidic environment helps kills the microbes coming from the rumen [53]. Additionally, Wang and co-authors reported decreased abomasum pH in ruminants as age increases [54]. All these observations mentioned above indicated that hydrochloric acid secretion might have decreased in the HE group, making the abomasum of the dosed young calves function more like an adult abomasum. For future follow-up studies, the changes of pH in abomasum should be tested in subsequent research along with the microbial diversity in the rumen with dosed calves.

Several genes encoding digestive enzymes showed significant expression changes in abomasum in the treated group. Chymosin (CYM) is produced by newborn ruminants in the lining of the abomasum to curdle the milk they ingest, thus allowing them to remain longer and be better absorbed [55]. A report has shown that the chymosin in abomasum declines with age [54]. In this study, *CYM* gene expression was significantly lower in the HE group (Table S1 log_2_FC = −5.64, *p* < 0.001). This indicated that milk protein digestion was decreased in the abomasum and the abomasum tissues were more mature in the HE group to adapt to rumen content artificial dosing. Notably, in the HE group, *PGA5* showed more than twofold decrease in expression (Table S1 log_2_FC = −2.78, *p* < 0.001). The *PGA5* encodes pepsinogen A, which is the proenzyme of pepsin and released by the chief cells in the stomach wall [56]. Pepsin is an endopeptidase that breaks down proteins and is one of the main digestive enzymes in the digestive systems of humans and many other animals [54]. This significant decrease in expression may be linked to the dosing from adult cow, affecting abomasal protein digestion. Bacteria in the rumen are considered more important in the production of microbial proteins than protozoan and fungal zoospores [57]. However, we found three lysozyme related genes (Table S1. *LYZ1*, log_2_FC = −4.77, *p* < 0.001; *LYZ2*, log_2_FC = −3.69, *p* < 0.001; *LYZ3*, log_2_FC = −4.93, *p* < 0.001) with significantly down-regulated expression changes in the HE group. Lysozyme is an antibacterial enzyme that digests bacteria present in the abomasum. It could lyse bacteria in the foregut, which are subsequently digested by digestive enzymes [58]. In the current study, significantly downregulated expression of *LYZ1, LYZ2*, and *LYZ3* implied that the digestion of bacteria decreased in the HE group of calves. In our companion study, we observed significant up-regulation of *SGPL1* [23]. *SGPL1* was reported to be essential for *Bacteroides* physiology, enabling them to survive in the intestine and to perform functions related to symbiosis [59]. Thus, the increased expression of *SGPL1* may indicate successful survival of exogenously dosed bacteria in the dosed calf. Collectively, the reduced expression of LYZ enzymes may imply that most artificially dosed microorganisms from the donor cow colonized in the rumen successfully, instead of being digested in the abomasum. On the other hand, lower lysozymes could reduce the activity and growth of rumen protozoa, which are one of the main causes of inefficient nitrogen use in cattle [60]. Dosing may be a potentially meaningful approach to improve the feed efficiency in cattle at an early age. A follow-up study may investigate the expression profiles of Lysozymes in rumen tissues with artificial dosing of adult rumen content in addition to investigating the colonization and abundance of protozoa.

Immunity-related genes responded to the dosing with elevated expression levels in the abomasum in the treated group. This finding suggests that calves respond to invasion of exogenous microorganisms by activating their immune and defense responses. Notably, interferon-stimulated gene 15 (*ISG15*) has anti-viral activity [61] and showed a more than 8-fold increase (Table S1 log_2_FC = 3.27, *p* < 0.001) in expression in the treated group. Radical S-adenosyl methionine domain containing 2, *RSAD2*, encoding viperin showed a four-fold change (Table S1 log_2_FC = 2.10, *p* = 0.0054). Interferon induced with helicase C domain 1 gene (*IFIH1*) encoded melanoma differentiation-associated protein 5, which functions as a pattern recognition receptor (recognizing dsRNA) that is a sensor for viruses [62]. All these implied a prominent defense response to viruses in abomasum tissue in the HE group. However, we did not observe clinical signs consistent with enteric infections or inflammation response in calves after dosing with adult cow rumen content. These observations likely depicted a picture of acting immune machinery, where the treated calves had a healthy balance of immune response to the exogenous dosing. Further work is needed to investigate the genes involved in inflammatory response to achieve a fuller picture of the impact of exogenous dosing on the host immune response and maturity in young calves.

We observed significant abundance increase in *Desulfovibrio*, which is a predominant genus in the rumen [63,64]. High-yielding dairy cows showed a significantly lower abundance of this genus compared to their low-yielding counter parts [65]. As we know, *Desulfovibrio* is a sulfate-reducing bacterium that metabolizes lactic and pyruvic acids to acetate and carbon dioxide, the latter of which can serve as an electron donor for sulfate reduction [66]. These changes could be adaptive responses to the acidic environment in abomasum. Although the abundance of *Desulfovibrio* was not significantly increased in the rumen as reported in our previous study [23], the significant increase of *Desulfovibrio* in the abomasum (Table 1) may be caused by a significantly acidic environment in the abomasum of treated calves. Lang and Wang reported decreased abomasum pH in ruminants as age increases [54]. It is worth further studying whether the maturity of abomasum is related to rumen development and the changes of rumen microorganisms. Furthermore, we found that *Desulfovibrio* was positively related to *TRPM4* (r > 0.7, *p* < 0.05, Figure 4), a gene involved in a variety of physiological and pathological processes, such as modulation of immune cell activity [41,67]. The tight control of Ca^2+^ influx by the *TRPM4* channel is critical for response to bacterial infection [68].

Another significantly changed genus is *Leptotrichia*, which represents anaerobic gram-negative rods of the phylum Fusobacteria. This genus was recently identified as one of the core microbes in rumen fluid in sheep [69]. Although they are difficult to identify or isolate, they have been shown to elicit immune responses in animal models and were considered as opportunistic pathogens, such as in hosts with neutropenia [70]. Some immunity-related cytokines mRNAs were significantly correlated with *Leptotrichia* species [71]. Additionally, *Leptotrichia* plays a crucial role in the transition from health to disease in piglets [72]. *Eubacterium* has tissue distribution specificity, as the relative abundance of *Eubacterium* was lower in the rumen compared with the reticulum (*p*-value = 0.027) [73] and was lower in rumen compared with the abomasum in our study (1102.84 ± 451.24 vs. 3362.17 ± 1081.49; *p* = 0.042). This genus had been reported as a beneficial bacteria [74] because of its ability to aid digestion, synthesize vitamins, suppress pathogenic microorganisms, and stimulate immune function. By arranging the abundance of species belonging to the *Eubacterium*, these species have been reported to be closely correlated with the age of the cow as well as milk production or inflammatory cytokines. Species in *Eubacterium* might help to advance milk production and mitigate inflammation [75]. 

Another interesting finding in our study is that we observed abundance variations in several genera between rumen and abomasum tissues. In our previous study in the rumen [23], these 3 genera (*Deinococcus, Leptotrichia*, and *Eubacterium*) were significantly lower in the HE group (Table 1, *p* < 0.05) consistent with our findings in the abomasum. Changes of *Tannerella* and *Eubacterium* were the opposite between rumen and abomasum after artificial dosing of rumen content from adult donor cow. *Tannerella* was higher in abomasum from the HE group compared to the control group, while lower in rumen in the HE group [23]. *Eubacterium* was higher in rumen in the HE group compared to the control group [23], while lower in abomasum. When we performed the association analysis, we identified negative correlation between *Deinococcus* and 3 URGs involved in immune system process (Figure 4, *TRPM4, TLR2, NLRX1*; r < −0.7, *p* < 0.05). *Leptotrichia* showed a negative association with *NLRX1*, which plays a role in host immunity during bacterial infections [76]. The log_2_FC of *TLR2* and *NLRX1* between two groups were significant (1.06 and 0.65, respectively, Table S1), suggesting that gene expression changes in these genes were part of the response in the host to adapt the dosing instead of the pathological infection.

For the longest time, the gut microbial community in cattle has been established primarily using rumen samples. The abomasum is largely untouched. As a traditional focus of the cattle GIT, the study in rumen only paints a portion of the stomach and the whole digestive system. In our study, the differences we observed in the two stomach chambers underline the importance of studying other compartments of the ruminant stomach. More studies in abomasum will help further understand the differential retention of dosed microbes and the functional impact of such differences.

## 5. Conclusions

Our study provided a transcriptome snapshot of the molecular mechanisms in the abomasum of young calves dosed with rumen content collected from adult cow. The genes encoding digestive proteins (*CYM* and *PAG5*) and functioning in bacteria digestion (*LYZ1, LYZ2,* and *LYZ3*) were down-regulated in abomasum after dosing. The ion transporters involved in HCl output of abomasum changed significantly. Specifically, genes with a role in pumping out of hydrogen ions (*ATP4A*) and chloride ions (*SLC26A7, SLC4A2,* and *CLIC6*) were down-regulated. Although 3 genes related to the response of virus (*ISG15, RSAD2,* and *IFIH1*) were up-regulated and changes of the microbial community in abomasum were highly correlated to some genes in immune response, calves dosed with adult cow rumen content did not appear to have clinical signs consistent with enteric infections. The differential abundance of several genera in the rumen and abomasum in our study further signifies the importance of a systematic investigation of all of the stomach chambers in the cattle. Such analysis will most likely propel comprehensive delineation of the complex interplay between the host and its microbial communities, and its role in cattle nutrition and health. Our results suggest that the dosing of adult rumen contents alters not only the composition of the active microorganisms in the abomasum, but also the molecular mechanisms in the abomasum tissue, including reduced protease secretion and decreased hydrochloric acid secretion. All in all, our study will help fill the molecular knowledge of abomasum in young calve, especially their transcriptome changes after early introduction of rumen content.

## Figures and Tables

**Table 1:** The active bacteria changes in abomasum identified by rRNA transcriptome analysis. HE: high efficiency; CON: sterilized rumen content.

Genus	Normalized Read Counts		SEM	*p*-Value
	Con	HE		
*Desulfovibrio*	1851.36	3369.71	168.04	0.021
*Tannerella*	617.13	2049.12	37.17	0.043
*Deinococcus*	40.74	0	1.16	0.047
*Leptotrichia*	111.58	30.45	2.58	0.042
*Eubacterium*	5175.98	1548.35	108.16	0.043

## Data Availability

The raw reads of abomasum samples were deposited in FigShare (doi: 10.6084/m9.figshare.12367589), the rRNA raw reads of abomasum tissues were submitted to NCBI with project accession number of PRJNA644386.

## Supplementary Materials

The following are available online at https://www.mdpi.com/2073-4425/12/3/424/s1,Table S1: Differentially expressed genes between HE and Con groups, Table S2: GO term annotation for the URGs and DRGs, Table S3: Genera in the abomasum between HE and Con groups. Table S4: The Pearson’s r value and *p*-value between the genera and the genes enriched in the immune response.
