# Supplementary material for: The Effects of Artificially Dosed Adult Rumen Contents on Abomasum Transcriptome and Associated Microbial Community Structure in Calves
Source: Genes (Basel). 2021 Mar 16;12(3):424. doi: 10.3390/genes12030424 (PMC7999174; doi:10.3390/genes12030424)
Supplement: Supplementary file 1 [file genes-12-00424-s001.zip › Supplementary Table 1-DEGs.pdf]

The effects of artificially dosed adult rumen contents on abomasum transcriptome and associated microbial community structure in calves

Naren Gaowa[1], Wenli Li[2]*, Brianna Murphy[2], Madison Cox[3]

1 State Key Laboratory of Animal Nutrition, Beijing Engineering Technology, Research Center of Raw Milk Quality and Safety Control, College of Animal Science and Technology, China Agricultural University, No.2 Yuanmingyuan West Road, Haidian, Beijing 100193, China.

2 The Cell Wall Utilization and Biology Laboratory, USDA Agricultural Research Service, US Dairy Forage Research Center, Madison, WI 53706, USA

3 Department of Microbiology, University of Wisconsin-Madison, Madison, WI, 53706, USA

*Correspondence: wenli.li@ars.usda.gov

Supplementary Table 1 Differentially expressed genes between HE and Con groups

Gene.Name	HE	Con	baseMean	log2FoldChange	pvalue
JAKMIP2	839.06	77.71	458.39	3.43	0.0000
ISG15	313.28	32.36	172.82	3.27	0.0006
BPIFA2A	306.73	32.21	169.47	3.24	0.0001
CCK	490.65	55.77	273.21	3.13	0.0200
ATP13A5	259.88	32.68	146.28	2.98	0.0312
OAS1X	620.02	102.76	361.39	2.59	0.0014
LOC100139670	313.63	58.38	186.00	2.42	0.0060
ADGRG7	1995.20	385.20	1190.20	2.37	0.0000
LOC781988	57.15	11.55	34.35	2.33	0.0010
LOC100298356	61.64	12.64	37.14	2.28	0.0240
IFI44	316.16	69.39	192.78	2.19	0.0045
C6H4orf17	44.17	9.46	26.81	2.19	0.0011
CFAP206	42.33	9.17	25.75	2.18	0.0072
PAX6	136.49	30.92	83.71	2.13	0.0011
IFIT3	125.72	28.58	77.15	2.12	0.0082
OAS1Y	1002.52	233.07	617.79	2.11	0.0012
RSAD2	895.31	208.47	551.89	2.10	0.0054
SLC28A3	32.77	7.54	20.15	2.04	0.0001
PCSK2	1397.96	348.94	873.45	2.00	0.0020
NEUROD1	54.68	14.15	34.41	1.95	0.0010
LYPD8	27.10	6.84	16.97	1.95	0.0003
FCGR2B	700.45	182.78	441.62	1.93	0.0035
SCTR	90.61	24.31	57.46	1.88	0.0015
CA8	25575.35	7082.50	16328.92	1.85	0.0045
LOC107131521	282.08	78.83	180.45	1.83	0.0013
ZBP1	746.80	212.77	479.78	1.81	0.0031
ISG12(B)	2313.07	662.02	1487.55	1.80	0.0064
AQP5	29.34	8.14	18.74	1.80	0.0438
SYT13	127.51	36.43	81.97	1.79	0.0007
RTP4	44.28	13.05	28.67	1.76	0.0324
C3H1orf168	63.00	18.02	40.51	1.76	0.0001
NPC1L1	123.98	36.51	80.25	1.75	0.0223
TMC5	218.40	64.36	141.38	1.75	0.0098
TXNDC8	55.11	16.98	36.04	1.75	0.0012
ISG20	185.95	55.38	120.66	1.74	0.0021
MX1	2410.85	724.91	1567.88	1.73	0.0071
FREM2	2098.10	632.42	1365.26	1.73	0.0000
EYA2	23.52	6.95	15.24	1.72	0.0321
BHMT2	48.13	14.68	31.40	1.72	0.0022
HRASLS	38.20	11.42	24.81	1.70	0.0055
SLC12A3	42.39	12.70	27.54	1.68	0.0002

APOL3	352.88	110.15	231.52	1.68	0.0005
GPR119	37.88	11.52	24.70	1.67	0.0105
CMPK2	440.85	140.39	290.62	1.65	0.0008
USP18	914.53	291.63	603.08	1.65	0.0013
EPSTI1	1475.52	473.38	974.45	1.64	0.0013
SST	3168.56	1025.40	2096.98	1.63	0.0208
OAS2	106.86	35.39	71.13	1.60	0.0144
PAPPA2	542.89	179.10	360.99	1.60	0.0381
CASR	152.78	50.72	101.75	1.58	0.0003
LPAR5	31.42	10.42	20.92	1.55	0.0038
CYP2B6	929.56	321.19	625.38	1.54	0.0001
FCGR2A	16.04	5.56	10.80	1.53	0.0022
LGALS9	1151.11	399.13	775.12	1.53	0.0000
TMPRSS4	285.79	98.96	192.37	1.52	0.0086
ACER2	1414.66	497.19	955.93	1.51	0.0055
PKIB	567.72	199.78	383.75	1.51	0.0000
OSR1	15.70	5.52	10.61	1.50	0.0441
IFI44L	346.87	122.56	234.72	1.50	0.0086
ATP2A3	3837.48	1390.54	2614.01	1.46	0.0037
KCNH3	14.43	5.19	9.81	1.46	0.0161
PAK6	34.17	12.86	23.52	1.46	0.0014
NME5	52.64	19.24	35.94	1.46	0.0021
SLC6A11	234.94	86.00	160.47	1.44	0.0198
PLCXD3	764.55	281.42	522.99	1.44	0.0055
LY86	192.93	70.99	131.96	1.44	0.0045
NR1I2	33.09	12.47	22.78	1.43	0.0166
DEGS2	213.76	80.07	146.91	1.42	0.0001
GPA33	27.57	10.31	18.94	1.41	0.0403
MCTP2	1681.83	633.79	1157.81	1.41	0.0029
CERKL	94.86	36.18	65.52	1.40	0.0033
LOC530929	7911.51	3022.18	5466.84	1.39	0.0163
DHX58	274.21	105.65	189.93	1.38	0.0022
MIR2904-3	31.81	12.66	22.24	1.36	0.0107
TSPAN3	2532.04	987.55	1759.80	1.36	0.0000
HERC6	1861.47	733.74	1297.61	1.34	0.0316
CCL16	147.06	58.68	102.87	1.33	0.0007
GBA3	52.49	21.79	37.14	1.33	0.0043
LDLRAD1	16.06	6.33	11.20	1.33	0.0216
KCNK5	898.47	358.57	628.52	1.33	0.0000
ANPEP	97.52	39.06	68.29	1.32	0.0003
HEPACAM2	541.95	218.10	380.02	1.32	0.0043
ARHGEF19	13.27	5.25	9.26	1.31	0.0387
BAIAP2L2	504.02	203.24	353.63	1.31	0.0000

KCNN4	499.56	200.98	350.27	1.31	0.0435
AMPH	159.27	64.67	111.97	1.31	0.0270
RASGRF1	30.30	12.28	21.29	1.30	0.0373
VWA3B	326.75	132.75	229.75	1.29	0.0020
XAF1	112.70	46.47	79.59	1.29	0.0260
UNC5CL	35.81	14.53	25.17	1.27	0.0472
B3GNT7	65.35	26.71	46.03	1.27	0.0329
HMGCS2	7606.70	3151.37	5379.04	1.27	0.0178
IGSF9	115.61	48.78	82.20	1.25	0.0146
ADCY1	47.34	20.50	33.92	1.24	0.0054
DGAT2	1161.70	494.43	828.06	1.23	0.0000
AMPD3	48.78	20.60	34.69	1.22	0.0191
MYH14	959.02	412.98	686.00	1.21	0.0011
MISP	621.15	267.52	444.33	1.21	0.0118
MEIS2	16618.32	7194.63	11906.48	1.21	0.0464
CA10	453.39	198.07	325.73	1.20	0.0271
EVPL	116.48	51.57	84.03	1.18	0.0067
PAH	52.00	23.47	37.74	1.18	0.0260
C16H1orf106	90.63	40.25	65.44	1.18	0.0001
F5	198.19	89.02	143.61	1.17	0.0209
ABHD1	329.50	147.05	238.28	1.16	0.0058
UGT1A6	4917.70	2200.33	3559.02	1.16	0.0121
LOC613966	404.03	181.85	292.94	1.16	0.0064
PCSK1	248.86	112.55	180.71	1.15	0.0016
CAPSL	61.57	28.05	44.81	1.15	0.0198
REP15	101.97	46.92	74.44	1.14	0.0075
GATM	3474.25	1578.91	2526.58	1.14	0.0221
SLC38A5	48.96	22.06	35.51	1.13	0.0068
TEX9	482.06	220.89	351.48	1.12	0.0203
FBP2	61.46	27.95	44.70	1.12	0.0416
CA13	957.68	440.88	699.28	1.12	0.0316
ABO	236.17	108.43	172.30	1.12	0.0063
SLC38A4	93.58	43.25	68.42	1.11	0.0005
OVOL1	122.82	56.87	89.85	1.11	0.0137
FAM102A	569.08	264.48	416.78	1.11	0.0004
MKRN2OS	29.60	13.51	21.55	1.10	0.0322
MYO7B	223.80	104.63	164.21	1.10	0.0261
MCF2L	124.45	58.44	91.44	1.10	0.0066
DAB1	203.67	94.83	149.25	1.10	0.0480
DTX3L	574.40	268.33	421.36	1.10	0.0011
GUCY2C	190.56	88.82	139.69	1.10	0.0065
MYOM3	14.52	6.82	10.67	1.09	0.0421
ECM1	263.95	124.24	194.09	1.08	0.0130

LDLRAD3	4218.13	1996.54	3107.34	1.08	0.0198
GCLC	980.95	465.48	723.21	1.08	0.0039
GPR63	55.24	26.24	40.74	1.07	0.0013
TLR2	36.04	16.77	26.40	1.06	0.0245
KCNB2	2373.15	1142.30	1757.72	1.06	0.0087
MAP3K15	643.12	309.57	476.35	1.05	0.0195
SLC35G2	178.72	86.14	132.43	1.05	0.0032
TWIST2	213.41	104.04	158.73	1.05	0.0065
CYP2C87	195.05	94.31	144.68	1.04	0.0271
MCOLN2	637.83	309.42	473.62	1.04	0.0134
AIM1L	138.64	67.41	103.02	1.04	0.0157
SORL1	2153.54	1047.69	1600.61	1.04	0.0197
IFIH1	802.31	390.48	596.40	1.04	0.0179
FAM83H	61.41	29.86	45.63	1.04	0.0391
AK8	169.67	83.46	126.57	1.04	0.0041
MST1R	335.37	164.13	249.75	1.03	0.0058
LOC514243	132.72	65.55	99.13	1.03	0.0187
GJB1	209.15	102.22	155.69	1.02	0.0244
UNC5B	562.77	276.86	419.81	1.02	0.0001
RORC	438.81	216.48	327.65	1.02	0.0378
ZNFX1	1466.63	724.07	1095.35	1.02	0.0215
MIR2904-1	180.73	90.02	135.38	1.02	0.0197
CAPN5	1820.63	902.25	1361.44	1.01	0.0010
HNF4A	671.59	334.47	503.03	1.00	0.0010
PARP14	1616.09	805.63	1210.86	1.00	0.0080
AGRN	418.41	209.50	313.96	1.00	0.0024
LOC505479	111.60	56.11	83.85	0.99	0.0421
SYTL2	20020.32	10061.30	15040.81	0.99	0.0174
LGALS3BP	456.54	230.08	343.31	0.99	0.0150
GDNF	113.97	57.58	85.78	0.99	0.0187
CNKSR1	55.66	28.20	41.93	0.99	0.0079
DAPP1	109.04	54.76	81.90	0.99	0.0149
GSDMB	158.79	80.40	119.59	0.98	0.0017
ITGB4	1262.18	639.38	950.78	0.98	0.0131
NCF2	24.28	12.40	18.34	0.97	0.0433
TRIM14	124.64	63.24	93.94	0.97	0.0026
LOC515578	155.24	79.38	117.31	0.97	0.0174
SEMA4G	1270.20	648.58	959.39	0.97	0.0028
TTLL10	336.68	172.43	254.56	0.97	0.0041
LPIN2	1444.94	742.38	1093.66	0.96	0.0015
NELL2	113.00	58.92	85.96	0.96	0.0407
RPH3AL	1011.59	522.19	766.89	0.95	0.0017
AK5	461.11	237.90	349.50	0.95	0.0076

AGBL3	67.44	34.93	51.19	0.95	0.0083
BMP7	481.39	249.26	365.32	0.95	0.0279
CHN2	5865.92	3043.15	4454.53	0.95	0.0013
DDX58	836.76	435.28	636.02	0.94	0.0268
MBD6	83.85	43.71	63.78	0.94	0.0210
ALDH1A1	5773.42	3007.92	4390.67	0.94	0.0282
NQO1	1590.83	830.91	1210.87	0.94	0.0183
DHRS13	24.05	12.76	18.41	0.93	0.0172
CCDC68	3218.87	1694.73	2456.80	0.93	0.0380
SULT2B1	63.66	33.49	48.57	0.92	0.0305
VILL	2631.73	1388.76	2010.25	0.92	0.0059
GPRC5B	509.04	268.16	388.60	0.92	0.0220
RIPK3	76.70	40.23	58.47	0.92	0.0180
FAM101A	165.14	86.76	125.95	0.92	0.0399
RASAL1	288.62	152.40	220.51	0.92	0.0177
EFNB1	165.92	87.75	126.84	0.91	0.0024
SWAP70	2144.80	1140.38	1642.59	0.91	0.0116
TMEM163	642.47	343.38	492.93	0.90	0.0066
ADAR	950.53	508.70	729.62	0.90	0.0136
PCTP	329.41	175.35	252.38	0.90	0.0136
BLNK	740.44	396.91	568.68	0.90	0.0148
RAMP1	194.00	103.50	148.75	0.90	0.0285
IGFBP2	614.37	330.05	472.21	0.90	0.0045
FAM83E	82.77	44.05	63.41	0.89	0.0077
ABCC3	2471.52	1331.05	1901.29	0.89	0.0016
AFMID	104.74	55.94	80.34	0.89	0.0005
MOV10	532.70	286.99	409.85	0.89	0.0068
PYCARD	76.67	41.00	58.83	0.89	0.0062
ANKRD29	275.26	149.04	212.15	0.89	0.0097
NECTIN4	103.87	56.16	80.01	0.88	0.0248
SLC22A23	804.51	436.07	620.29	0.88	0.0028
SYT17	856.68	465.25	660.96	0.88	0.0156
APOBEC3Z2	37.24	20.52	28.88	0.88	0.0227
DFNA5	55.23	29.64	42.43	0.88	0.0023
TBC1D16	2936.26	1597.92	2267.09	0.88	0.0207
CFAP69	400.92	218.33	309.62	0.87	0.0234
ITPR3	1359.38	743.49	1051.43	0.87	0.0113
SLC9A1	2983.14	1633.88	2308.51	0.87	0.0102
TTC22	1152.40	631.00	891.70	0.87	0.0424
FAM83G	100.61	55.01	77.81	0.87	0.0069
SGSM1	478.73	262.68	370.71	0.87	0.0083
GFRA2	293.80	161.25	227.52	0.87	0.0240
MDH1B	54.54	30.60	42.57	0.85	0.0177

PPARD	386.27	214.09	300.18	0.85	0.0025
PARP9	916.55	508.09	712.32	0.85	0.0065
ABCC6	49.29	28.05	38.67	0.85	0.0159
TMEM54	372.21	206.99	289.60	0.85	0.0182
PFKFB3	128.73	71.57	100.15	0.85	0.0005
LAD1	201.33	112.22	156.77	0.84	0.0258
TMEM79	162.49	91.08	126.78	0.84	0.0334
SMPD3	315.43	176.90	246.17	0.84	0.0253
PROSER2	510.80	286.14	398.47	0.84	0.0072
CD177	79.63	45.37	62.50	0.83	0.0377
SALL1	73.95	41.97	57.96	0.83	0.0334
FER1L6	9930.33	5612.21	7771.27	0.82	0.0433
ACE	105.24	59.52	82.38	0.82	0.0267
SPIRE2	108.33	61.25	84.79	0.82	0.0246
TRIM25	646.71	368.11	507.41	0.81	0.0029
TLL1	882.27	503.44	692.85	0.81	0.0094
CCDC189	24.35	13.99	19.17	0.81	0.0417
MOB3B	4561.27	2603.22	3582.24	0.81	0.0289
RGS14	49.79	28.78	39.29	0.81	0.0340
CNNM4	272.89	155.76	214.32	0.81	0.0033
ADAMTS5	412.85	235.66	324.25	0.81	0.0435
ADAP1	176.10	100.43	138.26	0.80	0.0066
TRANK1	2076.83	1190.73	1633.78	0.80	0.0211
ACOT7	186.86	106.67	146.77	0.80	0.0138
RPS6KA4	305.39	174.73	240.06	0.80	0.0323
IHH	682.82	392.71	537.76	0.80	0.0169
ICA1L	80.55	46.61	63.58	0.80	0.0234
PTPRH	241.04	138.57	189.81	0.80	0.0383
ABHD2	4994.79	2875.97	3935.38	0.80	0.0027
NCKAP5	152.21	87.52	119.87	0.79	0.0072
TRERF1	568.85	327.42	448.14	0.79	0.0244
SLC19A1	29.69	17.09	23.39	0.79	0.0420
CREB3L1	992.02	574.61	783.31	0.79	0.0048
SEMA4B	404.99	235.01	320.00	0.78	0.0020
SLC37A1	2715.52	1580.27	2147.89	0.78	0.0177
ADM	407.47	239.32	323.39	0.78	0.0002
PITX1	63.70	37.49	50.60	0.78	0.0395
PTPN3	1555.75	910.88	1233.32	0.77	0.0257
CCBE1	72.60	43.32	57.96	0.77	0.0241
SARDH	217.31	127.52	172.41	0.77	0.0065
KIAA1024L	29.09	16.88	22.99	0.76	0.0380
CABLES1	682.37	404.00	543.18	0.76	0.0023
MMP15	543.06	322.16	432.61	0.76	0.0089

TTC39A	871.36	516.74	694.05	0.75	0.0351
SYK	632.39	375.00	503.70	0.75	0.0056
SEZ6	36.42	21.74	29.08	0.75	0.0327
ARHGAP27	375.57	223.24	299.40	0.75	0.0114
CDCP1	1347.66	803.74	1075.70	0.75	0.0290
ARRDC4	431.05	256.23	343.64	0.75	0.0310
KHNYN	1138.80	678.41	908.60	0.75	0.0013
TRIM6	183.37	109.95	146.66	0.75	0.0309
HOMER2	717.98	428.07	573.02	0.74	0.0361
SP140L	684.11	409.60	546.85	0.74	0.0042
LOC512800	951.93	569.72	760.82	0.74	0.0117
VWA3A	34.16	20.84	27.50	0.74	0.0476
CTNNBIP1	127.07	76.16	101.61	0.74	0.0141
RAB11FIP4	150.40	90.03	120.21	0.74	0.0381
TXNRD1	1776.89	1067.14	1422.01	0.73	0.0131
ZFP36L1	877.04	527.61	702.32	0.73	0.0146
KDM1B	1093.32	658.52	875.92	0.73	0.0250
EQTN	54.78	32.50	43.64	0.73	0.0390
ETNK2	109.00	66.49	87.74	0.73	0.0360
GRTP1	181.12	109.63	145.38	0.72	0.0067
SLC6A6	171.45	104.28	137.86	0.72	0.0123
GGT7	33.99	20.96	27.47	0.72	0.0429
CHPT1	3397.52	2077.06	2737.29	0.71	0.0380
FBLIM1	1185.59	728.24	956.91	0.70	0.0020
CCDC85C	327.66	200.80	264.23	0.70	0.0093
PMVK	251.47	154.59	203.03	0.70	0.0148
FANCA	275.27	169.70	222.48	0.69	0.0424
PLBD1	71.35	44.08	57.71	0.69	0.0125
DISP1	964.17	596.31	780.24	0.69	0.0386
SRC	573.87	354.95	464.41	0.69	0.0033
KLHL13	380.40	235.22	307.81	0.69	0.0452
HS6ST1	299.93	186.57	243.25	0.69	0.0053
HHIP	731.76	454.48	593.12	0.69	0.0407
ERN2	2045.24	1271.93	1658.59	0.69	0.0285
CARD19	516.19	320.38	418.28	0.68	0.0063
LOC104975499	1937.80	1206.26	1572.03	0.68	0.0238
DQX1	204.41	127.94	166.17	0.68	0.0148
MERTK	669.01	418.72	543.87	0.68	0.0408
CHEK2	170.41	105.87	138.14	0.68	0.0186
DAPK2	989.57	619.65	804.61	0.67	0.0498
CANT1	72.33	45.02	58.68	0.67	0.0137
NR2F6	495.62	311.69	403.66	0.67	0.0046
SLC2A10	144.48	90.93	117.71	0.67	0.0451

TDP1	548.37	345.95	447.16	0.66	0.0157
LOC101902670	240.67	152.83	196.75	0.66	0.0407
IRF5	120.43	75.96	98.20	0.65	0.0200
FAM45A	840.36	534.63	687.49	0.65	0.0187
NLRX1	50.79	32.12	41.45	0.65	0.0468
MPP1	364.30	232.96	298.63	0.64	0.0273
MXD1	1270.40	814.62	1042.51	0.64	0.0398
ARID3A	315.98	204.16	260.07	0.64	0.0031
ATP11A	355.24	229.16	292.20	0.63	0.0209
BAI3	167.97	109.69	138.83	0.63	0.0422
FOSL2	2293.04	1486.53	1889.78	0.63	0.0112
TDRD7	2041.44	1328.56	1685.00	0.62	0.0312
EPHB1	55.31	35.81	45.56	0.61	0.0382
SYNJ2	620.00	405.43	512.72	0.61	0.0164
STK10	1705.08	1115.57	1410.33	0.61	0.0259
PLEKHA6	2373.50	1560.30	1966.90	0.61	0.0442
MAST3	168.83	111.41	140.12	0.60	0.0329
SIPA1L2	2336.05	1545.49	1940.77	0.60	0.0149
DLGAP4	1529.41	1012.12	1270.76	0.59	0.0073
LITAF	998.28	662.07	830.17	0.59	0.0423
ZBTB7A	98.62	65.71	82.17	0.59	0.0247
GRK5	4453.06	2965.56	3709.31	0.59	0.0028
ITGA3	1213.54	809.20	1011.37	0.58	0.0114
ST6GAL1	746.72	499.89	623.31	0.58	0.0025
SORD	219.42	147.91	183.66	0.57	0.0075
MDC1	169.09	113.24	141.16	0.57	0.0173
RIN2	906.68	612.59	759.63	0.56	0.0062
PML	356.22	241.63	298.93	0.56	0.0413
EVL	1604.08	1085.86	1344.97	0.56	0.0466
ELF4	330.07	224.37	277.22	0.56	0.0313
DOCK8	1163.54	789.59	976.56	0.56	0.0354
FRY	5383.96	3656.31	4520.13	0.56	0.0253
ELMSAN1	412.19	279.50	345.85	0.56	0.0088
POC1A	464.24	315.17	389.71	0.56	0.0291
TRPM4	867.68	592.47	730.07	0.55	0.0155
FBXL2	382.33	261.47	321.90	0.54	0.0389
PXMP4	106.79	73.58	90.18	0.54	0.0308
CD82	1255.77	867.44	1061.61	0.53	0.0259
ASIC2	157.56	109.48	133.52	0.53	0.0201
MACROD1	207.05	144.71	175.88	0.52	0.0078
RCBTB1	1129.11	788.85	958.98	0.52	0.0300
TBC1D22A	1123.85	785.23	954.54	0.52	0.0059
VANGL1	881.63	617.44	749.53	0.52	0.0296

ZMIZ1	2639.20	1846.24	2242.72	0.51	0.0177
WDR73	93.56	65.48	79.52	0.51	0.0425
SH3PXD2B	1826.44	1286.55	1556.49	0.51	0.0054
TMEM63B	942.81	662.99	802.90	0.51	0.0136
CCDC88C	1749.38	1232.10	1490.74	0.51	0.0113
KLF10	296.63	208.28	252.45	0.50	0.0411
NDUFV3	348.31	493.22	420.76	-0.50	0.0138
HSD17B12	1726.54	2444.01	2085.28	-0.50	0.0288
RFXAP	50.63	72.66	61.64	-0.50	0.0334
CCT2	990.30	1402.18	1196.24	-0.50	0.0076
MCFD2	234.44	332.85	283.65	-0.50	0.0151
GAB2	313.75	445.35	379.55	-0.50	0.0305
HIPK3	2037.49	2893.43	2465.46	-0.51	0.0402
EIF3I	512.53	729.39	620.96	-0.51	0.0202
PHAX	225.50	319.77	272.64	-0.51	0.0364
KIAA0141	228.88	326.82	277.85	-0.51	0.0234
CRBN	651.80	928.09	789.94	-0.51	0.0411
CHCHD3	4529.30	6448.17	5488.74	-0.51	0.0043
DYNLRB1	140.00	199.10	169.55	-0.51	0.0460
OAZ1	1015.30	1451.61	1233.45	-0.51	0.0174
UFSP2	412.06	588.65	500.35	-0.52	0.0171
COPS9	90.31	129.69	110.00	-0.52	0.0149
YBX1	2626.52	3763.23	3194.88	-0.52	0.0462
HIBADH	1345.79	1929.90	1637.85	-0.52	0.0316
PHF10	557.72	801.93	679.83	-0.52	0.0165
COQ6	157.60	227.62	192.61	-0.52	0.0301
SEC61B	292.99	422.46	357.73	-0.52	0.0147
PSMB6	193.82	277.94	235.88	-0.52	0.0357
HMGN4	136.63	196.49	166.56	-0.53	0.0293
PPIB	1058.42	1525.43	1291.93	-0.53	0.0300
ADGB	146.05	211.73	178.89	-0.53	0.0383
SUCO	1265.03	1828.93	1546.98	-0.53	0.0123
TMEM126A	87.46	126.24	106.85	-0.53	0.0101
JADE3	126.75	183.96	155.35	-0.53	0.0497
SPCS1	132.28	191.27	161.77	-0.54	0.0132
PRKAG1	138.94	201.71	170.33	-0.54	0.0022
APOO	119.59	174.51	147.05	-0.54	0.0222
SDCCAG3	90.44	130.40	110.42	-0.54	0.0343
SLC25A11	147.94	216.02	181.98	-0.54	0.0147
ATP6V0B	199.03	291.09	245.06	-0.54	0.0412
EIF1AX	400.04	582.42	491.23	-0.54	0.0003
HSPA9	1243.30	1813.04	1528.17	-0.54	0.0012
EIF2B4	129.54	188.71	159.13	-0.54	0.0050

MRPL51	180.32	262.97	221.64	-0.54	0.0182
MRPL27	107.07	156.85	131.96	-0.54	0.0491
SLC39A6	299.00	437.53	368.26	-0.55	0.0223
PABPC1	2498.99	3648.78	3073.88	-0.55	0.0270
ACY1	122.04	179.50	150.77	-0.55	0.0106
MARS	174.20	253.91	214.05	-0.55	0.0437
DLST	606.04	887.17	746.60	-0.55	0.0036
LOC785691	161.62	235.09	198.35	-0.55	0.0151
RPS27L	182.31	265.99	224.15	-0.55	0.0264
THYN1	69.97	102.20	86.09	-0.55	0.0132
RPL36	936.19	1377.32	1156.75	-0.56	0.0186
MRPL55	88.84	131.95	110.39	-0.56	0.0251
MRPS18B	67.30	100.69	84.00	-0.56	0.0439
PAIP1	804.70	1192.02	998.36	-0.57	0.0183
ZNF664	202.61	301.89	252.25	-0.57	0.0378
CCSER2	1504.40	2233.33	1868.86	-0.57	0.0457
ATF4	824.26	1223.09	1023.67	-0.57	0.0269
LAMTOR2	87.45	129.87	108.66	-0.57	0.0209
NDUFC2	352.39	526.84	439.61	-0.58	0.0019
LAMTOR5	101.52	150.99	126.25	-0.58	0.0102
PELI1	1295.99	1937.29	1616.64	-0.58	0.0262
HINT1	186.36	278.38	232.37	-0.58	0.0001
C4H7orf73	554.37	831.17	692.77	-0.58	0.0135
PABPN1	566.68	851.44	709.06	-0.58	0.0018
ST3GAL6	338.79	509.45	424.12	-0.58	0.0236
DNAJB9	182.91	274.63	228.77	-0.59	0.0245
IMMT	769.69	1155.22	962.46	-0.59	0.0007
SDHA	1037.63	1558.33	1297.98	-0.59	0.0436
IMPDH2	208.81	312.64	260.73	-0.59	0.0057
CNN3	1054.42	1584.98	1319.70	-0.59	0.0253
LARP4	727.07	1095.85	911.46	-0.59	0.0023
TMEM14C	260.79	394.13	327.46	-0.59	0.0003
CACYBP	291.00	437.62	364.31	-0.59	0.0031
MRPL35	94.72	143.61	119.17	-0.59	0.0189
WDR55	168.65	254.08	211.37	-0.59	0.0006
TMEM203	37.59	56.78	47.18	-0.59	0.0271
RPL36A	566.33	854.33	710.33	-0.60	0.0252
TACC1	2859.03	4319.93	3589.48	-0.60	0.0498
EID1	310.82	469.53	390.17	-0.60	0.0007
NDUFA6	259.23	393.19	326.21	-0.60	0.0384
PHB	326.26	495.35	410.81	-0.60	0.0239
MPC2	226.82	344.85	285.84	-0.60	0.0089
RPL36AL	456.43	691.07	573.75	-0.60	0.0090

MEA1	132.27	200.91	166.59	-0.60	0.0014
REXO2	297.05	450.54	373.79	-0.60	0.0126
SUPT4H1	118.03	180.56	149.30	-0.61	0.0016
TRAPPC3	108.66	166.64	137.65	-0.61	0.0046
SDF2	122.32	187.15	154.74	-0.61	0.0166
ABHD17B	529.90	808.59	669.24	-0.61	0.0333
MRPL49	106.08	161.67	133.87	-0.61	0.0055
ESD	419.80	641.70	530.75	-0.61	0.0065
NOL7	105.10	160.29	132.70	-0.61	0.0016
ZNF394	60.96	92.71	76.84	-0.61	0.0107
ATPIF1	273.96	418.32	346.14	-0.61	0.0228
AMOTL2	124.62	191.66	158.14	-0.61	0.0459
SUCLG1	1056.50	1619.23	1337.87	-0.61	0.0033
WDR45	137.58	211.78	174.68	-0.62	0.0478
DNAJB2	55.52	85.75	70.64	-0.62	0.0258
SORT1	584.52	897.79	741.15	-0.62	0.0126
UQCRC2	1296.41	1992.28	1644.34	-0.62	0.0020
POMGNT1	192.52	297.03	244.77	-0.62	0.0185
DFFA	136.04	209.30	172.67	-0.62	0.0024
GHITM	802.13	1234.31	1018.22	-0.62	0.0004
RAB12	229.68	353.01	291.35	-0.62	0.0376
SRP14	170.50	260.89	215.70	-0.62	0.0195
DLAT	254.47	393.28	323.88	-0.62	0.0021
JTB	99.49	152.43	125.96	-0.62	0.0025
NDUFV2	671.25	1035.49	853.37	-0.62	0.0081
MEMO1	1040.87	1606.93	1323.90	-0.63	0.0179
MAP3K5	3078.51	4749.43	3913.97	-0.63	0.0398
MTCH1	794.84	1228.62	1011.73	-0.63	0.0142
PIGC	255.55	394.68	325.12	-0.63	0.0104
TMEM143	37.83	59.45	48.64	-0.63	0.0381
SNX9	1156.22	1792.60	1474.41	-0.63	0.0124
PXMP2	119.76	186.25	153.00	-0.63	0.0348
FKBP4	406.68	631.30	518.99	-0.63	0.0054
MRPL11	117.53	181.92	149.72	-0.63	0.0027
BCO2	154.00	239.96	196.98	-0.63	0.0012
ATP6V1D	462.80	719.81	591.30	-0.64	0.0213
NDUFB8	328.69	511.76	420.23	-0.64	0.0015
KDR	1049.13	1633.21	1341.17	-0.64	0.0355
BEX2	100.99	156.65	128.82	-0.64	0.0484
IDH3A	304.88	476.49	390.69	-0.64	0.0030
LOC404103	120.73	188.03	154.38	-0.64	0.0457
CTPS1	188.70	295.03	241.86	-0.64	0.0005
COX1	157153.18	245499.98	201326.58	-0.64	0.0480

NDUFA12	282.02	442.29	362.15	-0.65	0.0093
ACAT1	828.68	1298.90	1063.79	-0.65	0.0313
PGAM1	616.29	965.93	791.11	-0.65	0.0161
FH	566.13	888.73	727.43	-0.65	0.0036
IKBIP	268.71	422.06	345.38	-0.65	0.0000
TMEM258	91.77	144.79	118.28	-0.65	0.0275
PRDX3	375.62	591.18	483.40	-0.65	0.0003
CHCHD2	384.17	603.63	493.90	-0.65	0.0000
MRPL53	134.67	211.28	172.97	-0.65	0.0036
HACD3	219.29	345.70	282.50	-0.65	0.0007
NAA20	410.10	646.12	528.11	-0.66	0.0001
MYH10	782.18	1234.13	1008.15	-0.66	0.0126
SGMS2	289.14	455.30	372.22	-0.66	0.0494
GPI	670.21	1058.31	864.26	-0.66	0.0014
PRSS23	656.90	1037.45	847.18	-0.66	0.0001
NDUFB3	208.92	330.62	269.77	-0.66	0.0044
NDUFA9	293.63	466.40	380.02	-0.66	0.0049
FAM96B	70.38	110.76	90.57	-0.66	0.0102
TMEM45A	526.83	838.15	682.49	-0.67	0.0006
TOMM40	72.49	115.40	93.94	-0.67	0.0271
ND4	14436.29	22964.93	18700.61	-0.67	0.0393
DNAJC19	106.05	169.64	137.84	-0.67	0.0102
COX7A2L	576.68	920.23	748.46	-0.67	0.0459
CUTA	122.40	196.42	159.41	-0.68	0.0353
TMCC3	186.26	297.78	242.02	-0.68	0.0300
NDUFB5	312.43	500.11	406.27	-0.68	0.0037
PRMT1	179.64	287.02	233.33	-0.68	0.0446
NDUFS1	1493.36	2393.59	1943.48	-0.68	0.0061
JDP2	188.79	303.15	245.97	-0.68	0.0406
ATP6V1E1	370.63	595.28	482.96	-0.68	0.0111
MRPL34	87.88	141.76	114.82	-0.69	0.0402
AFG3L2	403.04	650.32	526.68	-0.69	0.0012
TNIK	2720.36	4397.21	3558.78	-0.69	0.0144
VDAC2	865.70	1400.77	1133.24	-0.69	0.0001
VMA21	375.49	607.42	491.46	-0.69	0.0030
LDLRAD4	1032.48	1672.59	1352.54	-0.70	0.0285
SNHG12	45.38	74.85	60.12	-0.70	0.0416
DCUN1D4	703.52	1149.38	926.45	-0.71	0.0340
CCDC107	51.59	83.76	67.67	-0.71	0.0255
PDHA1	1319.83	2158.67	1739.25	-0.71	0.0127
IDH3B	395.46	647.88	521.67	-0.71	0.0160
FAHD1	49.94	81.70	65.82	-0.71	0.0368
SGPP1	141.21	232.64	186.92	-0.72	0.0025

BLOC1S1	53.11	87.43	70.27	-0.72	0.0303
TXN2	202.58	334.27	268.43	-0.72	0.0070
MRPL21	110.49	182.86	146.67	-0.72	0.0068
C1QBP	151.40	249.79	200.60	-0.72	0.0008
NDUFB11	129.61	214.01	171.81	-0.72	0.0145
C10H14orf166	647.76	1069.84	858.80	-0.72	0.0058
HADH	408.34	675.17	541.76	-0.72	0.0216
SLC25A10	84.62	140.89	112.76	-0.72	0.0168
PGK1	820.64	1357.06	1088.85	-0.73	0.0013
FGF13	47.54	78.83	63.18	-0.73	0.0041
MRPL16	73.02	121.51	97.27	-0.73	0.0201
TMEM35B	24.67	40.58	32.63	-0.73	0.0152
AK3	785.25	1300.52	1042.89	-0.73	0.0171
FKBP2	94.86	157.83	126.35	-0.73	0.0126
CAPRIN2	168.22	279.58	223.90	-0.73	0.0148
IL11RA	34.52	57.95	46.24	-0.73	0.0348
FAM189A2	326.07	542.63	434.35	-0.73	0.0080
SLIRP	126.23	210.51	168.37	-0.74	0.0000
TOMM40L	57.30	96.04	76.67	-0.74	0.0183
BSG	790.14	1320.03	1055.08	-0.74	0.0019
TOMM5	56.37	94.62	75.50	-0.74	0.0243
PTPN13	602.14	1006.56	804.35	-0.74	0.0074
SLC25A23	72.69	121.24	96.96	-0.74	0.0416
NDUFAB1	166.30	279.45	222.87	-0.74	0.0030
PDXK	116.24	195.67	155.95	-0.74	0.0473
ARSJ	396.60	666.50	531.55	-0.75	0.0328
LOC509972	25.46	42.39	33.93	-0.75	0.0134
SVBP	51.16	85.20	68.18	-0.75	0.0274
MPND	37.69	64.04	50.86	-0.75	0.0396
PLCG1	124.40	210.03	167.21	-0.75	0.0187
RNF34	389.57	658.58	524.07	-0.76	0.0353
STARD8	57.30	97.99	77.64	-0.76	0.0256
GABARAPL2	263.64	446.75	355.19	-0.76	0.0100
PEBP1	999.88	1694.45	1347.16	-0.76	0.0000
ATP5E	286.67	487.22	386.95	-0.76	0.0077
GADD45GIP1	22.01	37.84	29.92	-0.76	0.0407
DDT	45.97	79.20	62.58	-0.77	0.0181
ISCA1	334.83	571.27	453.05	-0.77	0.0273
TPI1	524.94	894.20	709.57	-0.77	0.0020
SPAG1	129.96	220.77	175.36	-0.77	0.0187
TIMM17A	51.93	89.33	70.63	-0.77	0.0295
CYCS	197.72	338.13	267.93	-0.77	0.0198
UBE2M	77.57	132.67	105.12	-0.77	0.0198

ND3	8269.47	14161.53	11215.50	-0.78	0.0073
PYCR2	38.91	67.37	53.14	-0.78	0.0349
ATP8	2555.69	4388.99	3472.34	-0.78	0.0057
MDH2	360.82	620.90	490.86	-0.78	0.0152
TMEM55A	102.89	176.92	139.90	-0.78	0.0158
COQ9	252.16	434.49	343.33	-0.78	0.0004
NDUFA1	109.55	190.05	149.80	-0.79	0.0015
ATP6	15797.37	27276.95	21537.16	-0.79	0.0129
NDUFA10	297.60	515.42	406.51	-0.79	0.0022
PANK1	328.57	568.03	448.30	-0.79	0.0002
PRDM5	1040.13	1801.43	1420.78	-0.79	0.0003
TOX	5576.08	9651.46	7613.77	-0.79	0.0155
LOC104976285	49.08	84.89	66.98	-0.79	0.0269
DMD	1864.94	3239.12	2552.03	-0.80	0.0040
FILIP1L	576.38	1000.82	788.60	-0.80	0.0081
HINT2	64.01	112.08	88.04	-0.80	0.0356
UQCRH	819.20	1429.43	1124.31	-0.80	0.0000
APPL1	427.95	748.45	588.20	-0.81	0.0018
AIFM1	453.67	795.29	624.48	-0.81	0.0376
VDAC3	399.45	699.33	549.39	-0.81	0.0111
PHF1	45.98	79.86	62.92	-0.81	0.0349
PKIA	163.56	287.77	225.67	-0.81	0.0400
OAT	185.57	326.58	256.07	-0.82	0.0201
LAMTOR1	72.35	128.10	100.23	-0.82	0.0309
HCCS	30.02	53.71	41.86	-0.82	0.0425
NREP	628.50	1108.54	868.52	-0.82	0.0429
PIN4	47.46	82.92	65.19	-0.82	0.0075
SMOX	229.37	406.12	317.75	-0.82	0.0351
TAF10	129.37	229.09	179.23	-0.82	0.0000
C9H6orf203	63.26	113.11	88.19	-0.82	0.0117
NDUFA2	92.02	163.57	127.79	-0.83	0.0124
NDUFC1	122.79	219.11	170.95	-0.83	0.0051
MBOAT1	496.43	882.83	689.63	-0.83	0.0061
CDKN1B	183.74	326.85	255.29	-0.83	0.0012
COX3	30140.85	53574.56	41857.70	-0.83	0.0058
MGC148714	477.31	851.80	664.56	-0.83	0.0000
IMP3	33.77	60.99	47.38	-0.84	0.0209
JMY	807.42	1445.80	1126.61	-0.84	0.0396
ACOT11	193.40	347.45	270.43	-0.84	0.0021
RDH14	54.35	97.85	76.10	-0.84	0.0007
C21H14orf2	201.63	363.52	282.57	-0.84	0.0005
ATP5J	326.28	586.28	456.28	-0.85	0.0000
KRTCAP2	110.89	200.21	155.55	-0.85	0.0013

SCAND1	38.90	70.65	54.77	-0.85	0.0151
MRPS36	133.57	242.21	187.89	-0.85	0.0017
ZDHHC14	595.59	1077.52	836.55	-0.85	0.0418
EMC6	23.53	42.08	32.80	-0.86	0.0276
ANK3	3398.05	6161.91	4779.98	-0.86	0.0145
BNIP3L	325.20	591.04	458.12	-0.86	0.0116
COX4I1	1005.41	1827.48	1416.44	-0.86	0.0023
KANK1	944.53	1724.62	1334.57	-0.87	0.0376
PAIP2B	43.31	79.16	61.23	-0.87	0.0020
SLMAP	2664.13	4869.81	3766.97	-0.87	0.0419
GPR34	11.55	21.15	16.35	-0.87	0.0377
NDUFA5	207.09	381.08	294.08	-0.87	0.0000
ATP5F1	1095.42	2016.51	1555.97	-0.88	0.0017
COX17	74.66	138.28	106.47	-0.88	0.0032
COX5A	566.14	1043.69	804.91	-0.88	0.0097
GBAS	750.38	1384.83	1067.61	-0.88	0.0000
SUOX	140.28	260.04	200.16	-0.88	0.0062
ARHGAP29	1424.96	2634.26	2029.61	-0.89	0.0013
PRDX5	196.92	365.56	281.24	-0.89	0.0156
POLR3K	53.09	98.84	75.97	-0.89	0.0010
ARHGAP24	2992.59	5549.76	4271.17	-0.89	0.0030
GNG5	458.51	853.17	655.84	-0.89	0.0080
ADCY10	25.47	48.61	37.04	-0.90	0.0136
POLDIP2	126.68	237.54	182.11	-0.90	0.0009
NDUFS2	559.43	1042.79	801.11	-0.90	0.0042
MBOAT2	76.45	142.87	109.66	-0.90	0.0170
ZNF169	28.53	53.90	41.22	-0.90	0.0486
C7H19orf70	91.77	172.20	131.99	-0.90	0.0070
ABI2	358.48	671.79	515.13	-0.90	0.0313
ISCU	222.76	418.03	320.40	-0.91	0.0050
NDUFS6	93.62	176.90	135.26	-0.91	0.0187
TBC1D8	535.54	1007.45	771.49	-0.91	0.0148
NDUFA8	167.07	315.83	241.45	-0.91	0.0012
UQCRB	367.99	696.56	532.28	-0.92	0.0006
PAQR3	52.27	99.00	75.64	-0.92	0.0382
NDUFB6	260.50	494.16	377.33	-0.92	0.0046
TIGD2	71.11	135.38	103.25	-0.92	0.0043
CITED2	79.31	150.19	114.75	-0.92	0.0370
ADK	2964.59	5624.78	4294.69	-0.92	0.0334
CYTB	11545.33	21961.03	16753.18	-0.93	0.0053
ATP5O	752.01	1432.60	1092.30	-0.93	0.0001
HECTD2	258.03	492.35	375.19	-0.93	0.0314
ZFYVE21	22.83	44.25	33.54	-0.94	0.0222

RDX	965.07	1851.27	1408.17	-0.94	0.0111
ATP5A1	3573.25	6858.72	5215.98	-0.94	0.0020
HSPB8	376.43	728.44	552.44	-0.95	0.0353
NDUFB10	193.37	375.16	284.26	-0.95	0.0001
APOLD1	47.07	90.74	68.91	-0.95	0.0390
EFHB	27.78	54.02	40.90	-0.95	0.0151
USMG5	207.92	404.81	306.36	-0.96	0.0000
CCDC92	76.03	149.00	112.51	-0.96	0.0315
KIT	366.34	713.65	540.00	-0.96	0.0490
TM4SF18	63.62	125.02	94.32	-0.96	0.0094
GOT2	454.92	888.90	671.91	-0.97	0.0003
PTPMT1	37.24	73.37	55.30	-0.97	0.0051
ILK	542.76	1061.59	802.17	-0.97	0.0469
SNAPIN	55.61	108.41	82.01	-0.97	0.0037
FAM20C	65.45	128.97	97.21	-0.97	0.0483
MMACHC	35.77	71.15	53.46	-0.97	0.0006
C1GALT1	1617.39	3177.67	2397.53	-0.97	0.0233
PRKACB	1319.43	2598.14	1958.78	-0.98	0.0049
SMDT1	116.97	231.47	174.22	-0.98	0.0055
SSSCA1	10.84	21.36	16.10	-0.98	0.0224
RNF149	454.72	898.83	676.77	-0.98	0.0305
PFDN4	75.15	147.99	111.57	-0.98	0.0482
ARSB	469.48	927.58	698.53	-0.98	0.0020
LOC100297725	30.51	61.30	45.90	-0.99	0.0069
HPRT1	200.89	397.97	299.43	-0.99	0.0344
ACADSB	314.32	623.34	468.83	-0.99	0.0036
FAM107A	62.89	125.64	94.27	-0.99	0.0241
SLC25A6	1682.87	3340.60	2511.74	-0.99	0.0034
MSN	1067.75	2121.85	1594.80	-0.99	0.0323
SOD1	323.04	644.59	483.81	-0.99	0.0006
PLAC8L1	26.00	52.36	39.18	-1.00	0.0266
COX7B	367.64	735.61	551.63	-1.00	0.0002
SELENOW	188.75	377.56	283.15	-1.00	0.0021
NDUFB1	71.04	143.20	107.12	-1.00	0.0030
DBT	661.22	1332.08	996.65	-1.01	0.0008
CYC1	364.65	734.82	549.73	-1.01	0.0368
ATP5C1	919.02	1853.26	1386.14	-1.01	0.0001
MYO5A	187.92	378.59	283.25	-1.01	0.0432
GCNT1	134.20	271.50	202.85	-1.01	0.0394
ROBO4	35.36	71.89	53.63	-1.01	0.0250
ND1	13225.19	26785.78	20005.49	-1.02	0.0094
PABPC4	489.95	993.40	741.68	-1.02	0.0120
UQCRFS1	467.88	949.29	708.59	-1.02	0.0009

GPR137C	38.93	79.17	59.05	-1.02	0.0115
ATP5H	750.99	1524.51	1137.75	-1.02	0.0001
CYP2U1	124.26	251.68	187.97	-1.02	0.0039
PHLDB2	1560.63	3171.56	2366.10	-1.02	0.0142
RGCC	68.43	139.72	104.07	-1.02	0.0121
PEMT	21.26	43.76	32.51	-1.03	0.0229
PDHB	260.72	532.73	396.73	-1.03	0.0001
AK1	58.34	120.51	89.42	-1.03	0.0059
ST3GAL4	137.45	283.44	210.44	-1.04	0.0007
TCEAL4	66.61	136.03	101.32	-1.04	0.0203
ROMO1	37.99	78.98	58.49	-1.04	0.0131
CCPG1	860.65	1772.83	1316.74	-1.04	0.0034
CCR5	6.26	13.35	9.81	-1.04	0.0448
PEA15	321.86	664.78	493.32	-1.05	0.0373
HSPE1	303.35	627.70	465.53	-1.05	0.0000
USH2A	11.49	23.67	17.58	-1.05	0.0296
C7H19orf60	25.04	52.36	38.70	-1.05	0.0056
DKK2	49.97	104.00	76.98	-1.06	0.0161
WIPI1	61.62	128.95	95.29	-1.06	0.0126
TIMM10	22.70	47.96	35.33	-1.06	0.0092
ACOX2	148.81	310.01	229.41	-1.06	0.0327
COX7C	330.73	690.27	510.50	-1.06	0.0070
SOD2	45.58	96.48	71.03	-1.07	0.0231
RASSF9	50.77	107.38	79.07	-1.07	0.0486
GOT1	199.23	419.57	309.40	-1.07	0.0032
NDUFA13	133.18	280.91	207.04	-1.07	0.0123
HSD17B10	151.01	318.71	234.86	-1.07	0.0001
MAF1	175.68	369.88	272.78	-1.07	0.0025
ZBTB16	4091.46	8624.04	6357.75	-1.08	0.0325
SEC14L1	343.59	725.37	534.48	-1.08	0.0249
ATP5J2	296.34	626.62	461.48	-1.08	0.0000
ETFRF1	139.69	296.25	217.97	-1.08	0.0131
DLD	803.61	1700.65	1252.13	-1.08	0.0039
CTSF	163.50	346.53	255.01	-1.08	0.0370
ATP5G3	509.27	1078.52	793.90	-1.08	0.0044
ABTB2	282.36	600.93	441.64	-1.09	0.0015
ESRRA	118.84	253.34	186.09	-1.09	0.0246
NDUFS3	167.85	357.83	262.84	-1.09	0.0025
RTN1	12.11	26.47	19.29	-1.09	0.0494
ERO1B	269.88	575.88	422.88	-1.09	0.0329
EPM2A	162.78	347.31	255.04	-1.09	0.0061
SLC9B2	50.47	108.73	79.60	-1.09	0.0094
COX6B1	136.71	292.75	214.73	-1.09	0.0007

APOBR	8.58	18.16	13.37	-1.09	0.0196
SMAD9	96.30	206.91	151.60	-1.09	0.0149
COX2	23655.54	50555.55	37105.55	-1.10	0.0038
ATP5L	553.52	1184.46	868.99	-1.10	0.0009
CYB5R1	22.97	49.77	36.37	-1.10	0.0081
PLCB1	11456.31	24627.09	18041.70	-1.10	0.0119
NR2F2	90.72	195.68	143.20	-1.11	0.0162
FBXO32	279.17	603.21	441.19	-1.11	0.0262
NDUFB9	203.10	440.81	321.96	-1.11	0.0002
ECI2	72.22	157.82	115.02	-1.12	0.0032
BTNL9	15.91	34.15	25.03	-1.12	0.0181
HSD17B8	56.28	123.10	89.69	-1.12	0.0015
IGIP	15.50	33.54	24.52	-1.12	0.0069
TCAIM	51.26	111.30	81.28	-1.12	0.0359
UQCR11	103.99	228.59	166.29	-1.13	0.0000
SLC35F3	549.44	1220.00	884.72	-1.15	0.0443
PM20D2	56.46	126.51	91.49	-1.15	0.0187
MAP1LC3A	44.35	99.46	71.91	-1.16	0.0072
PRDX2	603.94	1353.82	978.88	-1.16	0.0002
NPAT	699.84	1573.85	1136.85	-1.17	0.0072
MAGEF1	38.31	86.60	62.45	-1.17	0.0036
CDC42EP1	302.89	687.79	495.34	-1.18	0.0176
MDH1	770.44	1750.21	1260.33	-1.18	0.0002
NDUFS7	111.18	253.28	182.23	-1.18	0.0264
DYSF	419.62	956.23	687.93	-1.19	0.0382
TFRC	836.74	1911.27	1374.01	-1.19	0.0012
PCDH17	154.04	351.90	252.97	-1.19	0.0085
HEBP2	96.71	222.35	159.53	-1.20	0.0154
CLIC5	636.44	1464.53	1050.49	-1.20	0.0286
LRRC8B	154.74	356.92	255.83	-1.20	0.0398
ATP5B	3230.70	7452.42	5341.56	-1.21	0.0003
ARHGEF26	1031.92	2384.58	1708.25	-1.21	0.0020
TLE4	1194.56	2769.65	1982.10	-1.21	0.0097
UQCRC1	430.28	998.46	714.37	-1.21	0.0098
ATP5I	127.52	297.79	212.66	-1.22	0.0012
RUSC2	281.61	656.98	469.29	-1.22	0.0012
PDXP	13.99	32.38	23.18	-1.22	0.0086
ENPP4	654.33	1528.46	1091.40	-1.22	0.0349
RASGRP2	15.26	35.42	25.34	-1.23	0.0335
COX5B	572.04	1340.23	956.13	-1.23	0.0008
DZANK1	75.24	177.35	126.29	-1.24	0.0155
LOC782270	85.34	201.82	143.58	-1.24	0.0000
HMOX1	19.10	45.77	32.43	-1.24	0.0328

TMEM145	6.86	16.83	11.84	-1.25	0.0388
SYN3	70.33	166.80	118.57	-1.25	0.0478
COX7A2	192.51	460.49	326.50	-1.26	0.0011
BOLA1	9.95	24.08	17.01	-1.26	0.0120
EMILIN1	87.35	209.78	148.56	-1.27	0.0459
ROGDI	52.31	126.64	89.47	-1.27	0.0469
SMIM13	368.16	887.51	627.83	-1.27	0.0086
PTGDR	9.55	22.87	16.21	-1.27	0.0436
TRIM2	1944.32	4706.06	3325.19	-1.28	0.0167
CTH	32.50	79.71	56.11	-1.28	0.0124
AASS	539.02	1320.72	929.87	-1.29	0.0010
LYPD6B	63.38	156.64	110.01	-1.30	0.0296
RNF128	669.00	1647.83	1158.41	-1.30	0.0451
EPHA6	611.25	1509.98	1060.61	-1.30	0.0275
TMEM47	80.72	199.19	139.95	-1.30	0.0248
TMEM161B	1326.58	3277.38	2301.98	-1.30	0.0103
ZCCHC11	595.09	1472.90	1034.00	-1.31	0.0040
ST3GAL1	21.98	55.71	38.85	-1.32	0.0383
TRIB2	114.41	286.71	200.56	-1.32	0.0069
CS	1128.08	2819.79	1973.94	-1.32	0.0001
LOC783604	23.14	58.27	40.71	-1.33	0.0489
UQCC3	18.20	45.97	32.08	-1.33	0.0017
PLCG2	149.78	377.87	263.83	-1.33	0.0173
SIDT2	43.50	111.01	77.26	-1.34	0.0251
PPM1K	427.34	1084.78	756.06	-1.34	0.0002
PDLIM4	24.17	61.94	43.05	-1.36	0.0264
GPR176	172.49	443.02	307.75	-1.36	0.0210
VOPP1	342.08	878.61	610.34	-1.36	0.0027
SLC9A7	81.75	210.86	146.31	-1.36	0.0414
LOC783202	34.71	90.10	62.40	-1.37	0.0493
CCNG1	1581.70	4084.31	2833.00	-1.37	0.0019
DIXDC1	159.89	416.15	288.02	-1.38	0.0167
SLC25A3	1144.87	2993.27	2069.07	-1.39	0.0000
COX6A1	560.18	1465.10	1012.64	-1.39	0.0002
FIGN	17.89	47.12	32.51	-1.39	0.0080
PELI3	9.19	24.98	17.09	-1.40	0.0231
C11H9orf69	27.10	71.83	49.46	-1.41	0.0058
SMOC2	24.01	64.18	44.09	-1.41	0.0029
HCN1	24.76	66.07	45.41	-1.42	0.0023
SLC27A1	170.84	458.14	314.49	-1.42	0.0060
ZDHHC2	294.32	794.69	544.51	-1.43	0.0371
HTR4	21.06	57.76	39.41	-1.46	0.0331
PMEL	5.20	14.63	9.92	-1.48	0.0183

C22H3orf62	19.54	54.56	37.05	-1.48	0.0023
EPHA5	55.14	154.67	104.91	-1.49	0.0445
UQCR10	133.07	374.06	253.57	-1.49	0.0012
SNRNP25	17.55	49.65	33.60	-1.49	0.0000
STYXL1	13.44	37.90	25.67	-1.50	0.0374
PDGFD	1442.52	4090.87	2766.69	-1.50	0.0041
NKAIN2	19.30	55.64	37.47	-1.51	0.0052
RGMA	43.98	125.95	84.97	-1.52	0.0364
CD36	401.00	1153.66	777.33	-1.52	0.0286
BCAT2	150.46	438.53	294.49	-1.54	0.0061
DESI1	124.39	363.66	244.02	-1.54	0.0090
SLC7A6	146.43	429.19	287.81	-1.55	0.0000
NDUFA3	85.41	251.62	168.52	-1.56	0.0024
GMPR	66.62	196.01	131.32	-1.56	0.0282
SLC22A16	26.15	77.68	51.92	-1.56	0.0029
GPR155	92.51	273.84	183.17	-1.56	0.0111
CCDC60	52.32	155.52	103.92	-1.57	0.0106
FKBP5	3750.91	11159.51	7455.21	-1.57	0.0057
UQCRQ	111.78	336.44	224.11	-1.58	0.0001
TRPM3	181.40	545.43	363.42	-1.59	0.0141
PXYLP1	41.45	126.26	83.85	-1.60	0.0056
NDUFS8	47.96	146.46	97.21	-1.60	0.0053
ARMC12	25.70	78.37	52.03	-1.61	0.0203
CCDC8	65.64	200.26	132.95	-1.61	0.0187
PRELID2	510.03	1565.77	1037.90	-1.62	0.0041
PIK3C2G	8337.17	25722.22	17029.69	-1.63	0.0439
SLC30A4	93.89	290.51	192.20	-1.63	0.0040
GNA14	180.43	561.08	370.76	-1.64	0.0124
LGALS3	520.09	1621.27	1070.68	-1.64	0.0028
PLAUR	45.12	141.42	93.27	-1.64	0.0159
LRP2BP	5.17	16.61	10.89	-1.64	0.0154
LRRC63	9.86	31.55	20.71	-1.64	0.0175
OXCT1	770.35	2413.22	1591.78	-1.65	0.0008
STAP1	8.37	27.04	17.71	-1.65	0.0210
RAB3C	90.02	286.80	188.41	-1.67	0.0143
METTL12	15.11	48.80	31.95	-1.67	0.0021
STBD1	17.86	56.63	37.24	-1.68	0.0262
GLUL	2054.97	6611.76	4333.36	-1.69	0.0079
CTNND2	36.93	119.53	78.23	-1.69	0.0082
OPLAH	16.79	54.82	35.80	-1.70	0.0334
ESM1	23.06	76.53	49.80	-1.72	0.0210
LURAP1	15.30	50.90	33.10	-1.72	0.0118
RANGRF	7.37	24.53	15.95	-1.73	0.0007

PPP1R9A	762.16	2528.92	1645.54	-1.73	0.0015
CBLN2	7.61	24.71	16.16	-1.73	0.0040
NKIRAS1	23.00	77.41	50.20	-1.74	0.0003
NALCN	71.16	240.11	155.63	-1.75	0.0187
LOXL4	6.32	20.90	13.61	-1.76	0.0323
CDH18	61.55	212.80	137.18	-1.78	0.0110
RALYL	48.44	167.10	107.77	-1.79	0.0429
ENTPD6	103.76	360.16	231.96	-1.79	0.0003
CHCHD10	43.61	151.80	97.71	-1.79	0.0034
NPR3	59.97	209.20	134.59	-1.80	0.0186
VEPH1	137.77	480.40	309.09	-1.80	0.0082
SLC25A5	427.39	1496.24	961.82	-1.81	0.0025
SATB1	382.60	1339.27	860.94	-1.81	0.0015
MYZAP	609.39	2136.25	1372.82	-1.81	0.0001
FZD7	13.32	47.91	30.61	-1.85	0.0134
ZNF385B	236.15	851.38	543.77	-1.85	0.0308
SLC25A30	304.30	1099.44	701.87	-1.85	0.0008
SLC2A4	22.80	82.40	52.60	-1.86	0.0366
LIFR	794.65	2890.10	1842.38	-1.86	0.0049
ATP7B	210.75	767.79	489.27	-1.86	0.0085
CLGN	11.81	42.79	27.30	-1.87	0.0014
IL20RA	312.61	1149.28	730.94	-1.88	0.0179
C5AR2	10.36	38.61	24.49	-1.88	0.0150
MYCBPAP	24.93	92.26	58.60	-1.88	0.0412
FAM149A	144.10	534.45	339.28	-1.89	0.0014
PDZD2	221.96	837.64	529.80	-1.92	0.0024
NDUFA4	293.67	1123.88	708.77	-1.94	0.0001
GPRIN3	31.37	120.97	76.17	-1.94	0.0361
AUTS2	72.35	280.10	176.22	-1.95	0.0007
LOC505383	83.58	324.26	203.92	-1.96	0.0009
CA9	352.50	1370.62	861.56	-1.96	0.0152
PLEKHB1	216.85	846.41	531.63	-1.96	0.0089
DIRAS2	12.75	50.09	31.42	-1.97	0.0425
CA11	6.41	25.79	16.10	-1.97	0.0080
MATK	15.63	61.89	38.76	-1.97	0.0126
ME3	202.30	800.74	501.52	-1.98	0.0023
CIART	7.73	30.70	19.21	-1.98	0.0165
AHCYL2	757.34	3003.64	1880.49	-1.99	0.0005
CKMT2	17.67	72.03	44.85	-2.00	0.0008
PBLD	7.87	32.04	19.96	-2.01	0.0481
SLC16A10	551.91	2251.12	1401.52	-2.03	0.0053
RBFOX3	15.85	64.71	40.28	-2.03	0.0105
SNN	7.94	33.10	20.52	-2.04	0.0269

TIAM2	234.75	988.24	611.50	-2.07	0.0004
KLF15	24.65	105.19	64.92	-2.09	0.0033
IDH2	682.85	2959.06	1820.95	-2.12	0.0001
OTOGL	8.98	40.19	24.58	-2.17	0.0396
FRZB	27.95	125.45	76.70	-2.17	0.0474
ITIH4	43.66	198.43	121.05	-2.18	0.0007
SMARCD3	81.75	374.02	227.89	-2.19	0.0001
MBNL3	75.06	346.68	210.87	-2.21	0.0015
NNT	1412.89	6523.66	3968.28	-2.21	0.0001
SLC22A3	294.44	1363.12	828.78	-2.21	0.0000
CPT1B	14.00	64.80	39.40	-2.21	0.0009
RASGRP3	149.26	712.51	430.89	-2.25	0.0015
RASGRP1	79.08	387.55	233.31	-2.29	0.0050
EGLN3	101.59	500.95	301.27	-2.30	0.0046
CDKN2B	8.61	43.28	25.95	-2.33	0.0132
ATP5G1	167.18	845.47	506.32	-2.34	0.0002
CKB	352.14	1805.26	1078.70	-2.36	0.0001
SLC7A8	34.69	178.67	106.68	-2.36	0.0100
SLC4A2	113.26	592.63	352.95	-2.39	0.0028
SCN1B	12.08	64.09	38.09	-2.39	0.0008
CISD3	7.22	38.62	22.92	-2.40	0.0001
CADM4	5.24	28.14	16.69	-2.42	0.0046
DTNA	1092.91	6051.76	3572.34	-2.47	0.0000
LOC100196897	5.84	33.08	19.46	-2.49	0.0095
KCTD16	61.79	352.22	207.00	-2.51	0.0027
IGLL1	152.28	877.86	515.07	-2.53	0.0130
OPCML	43.80	253.26	148.53	-2.53	0.0077
MAPK13	49.60	297.85	173.72	-2.58	0.0036
SLC1A2	62.36	376.58	219.47	-2.59	0.0487
ANKRD55	5.31	32.83	19.07	-2.62	0.0106
PCNX2	110.83	680.07	395.45	-2.62	0.0026
CHST9	60.50	388.29	224.39	-2.68	0.0036
DNAH5	9.02	58.62	33.82	-2.68	0.0012
UNC13D	10.64	70.65	40.64	-2.72	0.0019
RNF212B	31.58	215.39	123.49	-2.77	0.0048
PGA5	25905.36	178278.37	102091.86	-2.78	0.0008
PPARGC1A	413.25	2893.83	1653.54	-2.81	0.0000
LDHB	380.23	2829.40	1604.82	-2.90	0.0000
PTGER3	304.39	2274.86	1289.63	-2.90	0.0000
SERPINA5	108.59	812.39	460.49	-2.90	0.0037
TMED6	14.56	114.33	64.45	-2.97	0.0007
PALM3	8.14	65.39	36.77	-2.99	0.0024
ASAH2	8.26	70.91	39.58	-3.09	0.0014

NXPE2	15.23	140.46	77.85	-3.20	0.0021
SLC16A7	670.19	6400.76	3535.48	-3.26	0.0000
RBM46	11.22	108.68	59.95	-3.28	0.0020
FAM20A	178.11	1878.46	1028.28	-3.40	0.0002
GABRB1	35.73	413.88	224.81	-3.53	0.0008
COX8B	26.62	337.86	182.24	-3.67	0.0002
CLIC6	160.61	2068.36	1114.48	-3.69	0.0068
LYZ2	50879.80	655257.22	353068.51	-3.69	0.0003
SLC25A4	95.20	1257.29	676.24	-3.72	0.0000
ACACB	15.72	208.96	112.34	-3.72	0.0006
BMPR1B	24.25	342.32	183.28	-3.82	0.0000
ATP6V1G3	10.40	160.94	85.67	-3.95	0.0123
FABP3	28.43	443.60	236.02	-3.96	0.0000
GHRL	77.61	1259.19	668.40	-4.02	0.0064
COL17A1	27.60	483.37	255.48	-4.13	0.0015
SERPINI1	42.34	744.96	393.65	-4.14	0.0014
EIF5A2	21.47	441.25	231.36	-4.36	0.0000
GIMD1	7.56	200.80	104.18	-4.72	0.0000
LYZ1	8713.19	237176.25	122944.72	-4.77	0.0008
LYZ3	3162.47	96643.75	49903.11	-4.93	0.0006
HDC	10.66	331.31	170.99	-4.95	0.0000
FLT3	25.39	902.83	464.11	-5.15	0.0003
CYM	4056.21	201922.11	102989.16	-5.64	0.0007
CDH16	21.25	1073.80	547.52	-5.66	0.0001
SLC26A7	35.17	2363.22	1199.19	-6.07	0.0000
AQP4	8.59	853.15	430.87	-6.63	0.0002
KCND2	25.08	3123.41	1574.24	-6.96	0.0000
ESRRG	115.75	16967.51	8541.63	-7.20	0.0000
ATP4A	97.51	33999.30	17048.41	-8.45	0.0000
LOC516849	8.06	3855.18	1931.62	-8.90	0.0000
LOC107131263	15.32	8001.66	4008.49	-9.03	0.0000
