# Supplementary material for: The Effects of Artificially Dosed Adult Rumen Contents on Abomasum Transcriptome and Associated Microbial Community Structure in Calves
Source: Genes (Basel). 2021 Mar 16;12(3):424. doi: 10.3390/genes12030424 (PMC7999174; doi:10.3390/genes12030424)
Supplement: Supplementary file 1 [file genes-12-00424-s001.zip › Supplementary Table 2-GO.pdf]

The effects of artificially dosed adult rumen contents on abomasum transcriptome and associated microbial community structure in calves

Naren Gaowa[1], Wenli Li[2]*, Brianna Murphy[2], Madison Cox[3]

1 State Key Laboratory of Animal Nutrition, Beijing Engineering Technology, Research Center of Raw Milk Quality and Safety Control, College of Animal Science and Technology, China Agricultural University, No.2 Yuanmingyuan West Road, Haidian, Beijing 100193, China.
2 The Cell Wall Utilization and Biology Laboratory, USDA Agricultural Research Service, US Dairy Forage Research Center, Madison, WI 53706, USA
3 Department of Microbiology, University of Wisconsin-Madison, Madison, WI, 53706, USA
*Correspondence: wenli.li@ars.usda.gov

Supplementary Table 2. GO term annotation for the URGs and DRGs

Category	Term	Count	%	FDR	Genes
GOTERM_BP.	GO:0002376~immune system process	26	7.69	0.0000	TRPM4, IFIH1, FFAR3, LY86, HERC6, PML, TLR2, RSAD2, NLRX1, TRIM25, PADI4, OAS2, SRC, LGALS9, ISG20, DDX58, IFIT3, KCNN4, IRF5, PYCARD, MST1R, MX1, DHX58, ZBP1, ADAR, SYK
GOTERM_BP.	GO:0045087~innate immune response	24	7.10	0.0004	IFIH1, ELF4, LY86, HERC6, TLR2, TRIM14, PML, RSAD2, NLRX1, TRIM25, PADI4, OAS2, SRC, ISG20, IFIT3, DDX58, IRF5, PYCARD, MST1R, MX1, DHX58, SYK, ADAR, ZBP1
GOTERM_BP.	GO:0051607~defense response to virus	15	4.44	0.0012	IFIH1, PML, RSAD2, TRIM25, OAS2, ISG20, IFIT3, DDX58, IRF5, ISG15, PYCARD, MX1, DHX58, ADAR, ZBP1
GOTERM_BP.	GO:0009615~response to virus	11	3.25	0.0020	IFIT3, DDX58, IFIH1, RSAD2, MST1R, OAS2, MX1, SRC, DHX58, ISG20, ADAR
GOTERM_CC.	GO:0016020~membrane	167	49.41	0.0000	CASR, ADCY1, TSPAN3, REP15, LYPD8, NELL2, TLR2, AQP5, SCTR, DAB1, UNC5B, CREB3L1, GNG3, MCOLN2, MX1, HTR1E, SYK, CLCA2, NCF2, VANGL1, GATM, EFNB1, CDHR2, SLC22A23, UNC5CL, SPIRE2, CHPT1, SGSM1, F5, LPAR5, HRASLS, CCR3, SLC37A1, PARP14, RIPK3, MST1R, IGSF9, FBXL2, SLC38A5, SLC38A4, SLC2A10, RTP4, LITAF, STK10, ITGB4, OAS2, EPHB1, SRC, TMEM63B, ACE, LGALS3BP, B3GNT7, CNR2, AGRN, TMEM79, BLNK, SLC28A3, ST6GAL1, PTPN3, KCNB2, SLC6A11, ACER2, ATP11A, EVL, ITGA3, DOCK8, ADGRG7, GUCY2C, HOMER2, ATP13A5, DDX58, GGT7, P2RX1, PARP9, ATP2A3, FREM2, SLC35G2, SYT13, SYTL2, MYH14, GRK5, MERTK, ABO, PDZD3, SYT17, SLC9A1, ADAR, SORL1, LRRC19, CDCP1, ANPEP, PMVK, MCF2L, CANT1, TMEM54, MCTP2, PXMP4, NPC1L1, SYNJ2, HHIP, RAMP1, IHH, TRPM4, GAPT, MPP1, RPH3AL, ARHGAP27, HEPACAM2, NECTIN4, ARRDC4, MMP15, SLC3A1, LPIN2, LDLRAD3, RAB11FIP4, MAST1, CHRM5, SEMA4G, DGAT2, CD82, SEMA4B, CARD19, GPA33, ERN2, EQTN, SEZ6, KCNH3, DEGS2, SORD, FFAR3, GPR63, PML, NLRX1, RSAD2, ABHD2, ABHD1, SLC19A1, GPRC5B, AMPH, PCSK2, DAPP1, DISP1, CD177, HS6ST1, DFNA5,

CNNM4, GPR119, BAIAP2L2, SLC12A3, DHRS13, SWAP70, TMC5, DLGAP4, ASIC2, ITPR3, GJB1, RGS14, TMPRSS4, KCNN4, FCGR2B, TMEM163, SLC6A6, ABCC3, CHN2, SMPD3, GFRA2, ABCC6

GOTERM_CC.	GO:0005737~cytoplasm	145	42.90	0.0032	CASR, ADCY1, NELL2, DQX1, TLR2, CALB1, ISG20, RCBTB1, WNT4, WDR73, ISG15, MX1, NQO1, PITX1, TWIST2, SYK, MDH1B, NCF2, DTX3L, EFNB1, PCTP, UNC5CL, MLXIPL, IFI44, PKIB, FBP2, SPIRE2, FRY, TNNT2, SGSM1, HNF4A, RASGRF1, TXNDC8, HRASLS, PARP14, RIPK3, IFIH1, SLC2A10, RTP4, LITAF, TDRD7, STK10, ITGB4, OAS2, EPHB1, SRC, ADAP1, RASAL1, MOV10, ACE, MEIS2, AGRN, DHX58, TMEM79, BLNK, AGBL3, BHMT2, PTPN3, SLC6A11, ARID3A, EVL, AK5, MISP, HOMER2, LGALS9, AK8, DDX58, POC1A, PARP9, SALL1, SYTL2, MYH14, GRK5, MERTK, PDZD3, SLC9A1, ADAR, MYO7B, PAX6, LRRC19, SULT2B1, IFI44L, ANPEP, PMVK, MCF2L, AFMID, PAK6, ACOT7, MCTP2, CERKL, TRIM6, SYNJ2, HHIP, FAM83H, FAM83G, FANCA, CABLES1, SH3PXD2B, CCDC88C, CAPSL, HERC6, RPH3AL, ARHGAP27, FBLIM1, HEPACAM2, PADI4, LPIN2, DAPK2, CTNNBIP1, MAST1, EYA2, TTLL10, EVPL, ADM, ZMIZ1, RIN2, HIPK4, TXNRD1, EQTN, SEZ6, PML, AMPH, ZFP36L1, ALDH1A1, DAPP1, MYOM3, PYCARD, XAF1, ZBP1, DFNA5, CAPN5, SWAP70, TRIM25, ITPR3, GJB1, RGS14, IFIT3, RPS6KA4, IRF5, FCGR2B, TDP1, CFAP206, NEUROD1, CHN2, SMPD3
GOTERM_MF.	GO:0008137~NADH dehydrogenase (ubiquinone) activity	19	2.95	0.0000	NDUFA4, NDUFA5, ND1, NDUFA2, NDUFA8, ND4, NDUFB8, NDUFB9, ND3, NDUFC2, NDUFA13, NDUFA10, NDUFA12, NDUFS7, NDUFV2, NDUFS8, NDUFS3, NDUFS2, NDUFS1
GOTERM_MF.	GO:0004129~cytochrome-c oxidase activity	14	2.18	0.0000	NDUFA4, COX7A2, COX3, COX8B, COX2, COX1, COX7B, COX6B1, COX7C, COX4I1, COX6A1, COX7A2L, COX5A, COX5B
GOTERM_MF.	GO:0016491~oxidoreductase activity	54	8.40	0.0000	ACOX2, LDHB, CYB5R1, PRDX5, PRDX2, PRDX3, UQCRFS1, PDHB, HIBADH, NDUFS7, HMOX1, NDUFS8, LOXL4, SMOX, PDHA1, HADH, NDUFS3, ATP8, NDUFS2, IMPDH2, NDUFS1, SUOX, ND1, ND4, AIFM1, ND3, GMPR, COQ6, PYCR2, NNT, DLD, MDH2, MDH1, CYP2U1, HSD17B10, ME3, ACADSB, HSD17B12, EGLN3, CYTB, AASS, MMACHC,

					IDH2, SOD1, IDH3A, SOD2, SDHA, COX2, UOX, COX1, TDH, NDUFV2, RDH14, BCO2
GOTERM_MF.	GO:0003954~NADH dehydrogenase activity	10	1.56	0.0000	NDUFS7, ND1, NDUFA9, NDUFB8, NDUFS8, NDUFV2, NDUFA13, NDUFS3, NDUFS2, NDUFS1
GOTERM_MF.	GO:0046933~proton-transporting ATP synthase activity, rotational mechanism	10	1.56	0.0000	ATP5E, ATP5B, ATP5F1, ATP5C1, ATP5L, ATP5O, ATP5A1, ATP5G1, ATP5H, ATP5G3
GOTERM_MF.	GO:0008121~ubiquinol-cytochrome-c reductase activity	7	1.09	0.0000	UQCR10, UQCR11, UQCRH, CYTB, UQCRFS1, UQCRQ, UQCRB
GOTERM_MF.	GO:0003824~catalytic activity	39	6.07	0.0002	UQCRC2, ETNPPL, ARSB, SLC27A1, LDHB, BCAT2, UQCRC1, HINT1, HINT2, ENPP4, ARSJ, PGAM1, AASS, ACAT1, PDHB, CKB, FAHD1, GOT2, TPI1, GOT1, OPLAH, SYN3, HDC, CKMT2, PIGC, IMPDH2, ECI2, LYZ2, LYZ1, SUCLG1, ACACB, GMPR, GLUL, CTH, PPM1K, TDH, OAT, MDH2, MDH1
GOTERM_MF.	GO:0009055~electron carrier activity	12	1.87	0.0009	ACOX2, SDHA, ACADSB, UQCR11, COX7A2, COX1, CYC1, CYCS, CYTB, COX7A2L, NDUFA12, NDUFS1
GOTERM_MF.	GO:0051287~NAD binding	12	1.87	0.0013	LDHB, HSD17B10, ME3, NNT, DLD, IDH2, IDH3B, AHCYL2, NDUFS2, IDH3A, HIBADH, MDH1
GOTERM_MF.	GO:0015078~hydrogen ion transmembrane transporter activity	9	1.40	0.0070	ATP5F1, ATP5L, ATP5G1, ATP5H, ATP5G3, ATP8, ATP6V0B, ATP6, ATP5J
GOTERM_MF.	GO:0016651~oxidoreductase activity, acting on NAD(P)H	6	0.93	0.0291	NDUFA5, AIFM1, NDUFS8, NDUFS3, NDUFS2, NDUFS1

GOTERM_BP.	GO:0055114~oxidation-reduction process	86	13.37	0.0000	UQCRC2, MRPS36, ACOX2, LDHB, CYB5R1, UQCRC1, CYC1, NDUFAB1, PRDX5, PRDX2, PRDX3, UQCRFS1, UQCRQ, HIBADH, PDHB, NDUFS7, NDUFS6, UQCR10, UQCR11, HMOX1, NDUFS8, SMOX, LOXL4, PDHA1, NDUFS3, HADH, NDUFS2, IMPDH2, NDUFS1, ND1, SUOX, NDUFB11, NDUFB10, ND4, AIFM1, ND3, CYCS, NDUFC2, NDUFC1, GMPR, NDUFA10, NDUFA12, COQ6, PYCR2, NNT, UQCRH, DLD, MDH2, MDH1, UQCRB, NDUFB3, CYP2U1, HSD17B10, NDUFB5, ACADSB, ME3, NDUFB6, TXN2, NDUFB8, NDUFB9, HSD17B12, EGLN3, CYTB, AASS, MMACHC, IDH2, NDUFA4, NDUFA5, NDUFA2, NDUFA3, NDUFA8, NDUFA9, NDUFA6, SOD1, NDUFA1, IDH3A, SOD2, NDUFV3, SDHA, COX2, UOX, COX1, TDH, NDUFV2, BCO2, RDH14
GOTERM_BP.	GO:0006810~transport	136	21.15	0.0000	SLC22A16, SLC9A7, GABRB1, SLC7A8, NDUFAB1, AQP4, SLC7A6, GOT2, UQCR10, UQCR11, SLC2A4, SLC25A23, SLC9B2, SLC4A2, NALCN, ATP8, ATP6, KCND2, SLC25A30, SEC61B, CD36, SLC25A35, ATP5C1, RAB12, ATP6V1G3, DYNLRB1, TRAPPC3, SNX9, SCN1B, TXN2, ATP6V0B, OAZ1, TOMM5, MTCH1, APOBR, SLC30A4, SLC35F3, HCN1, HCN2, GABARAPL2, ATP5J2, TOMM40, MPC2, VDAC2, VDAC3, ABCB5, NDUFV3, SLC25A11, COX2, COX1, SLC25A10, TOMM40L, NDUFV2, FABP3, ATP7B, UQCRC2, KCNJ16, SLC27A1, ATP5E, UQCRC1, SLC16A10, LRRC8B, ATP5B, TIMM17A, CYC1, UQCRFS1, UQCRQ, NDUFS7, NDUFS6, SLC1A2, NDUFS8, SLC25A3, ATP5L, SLC22A3, ATP5O, NDUFS3, NDUFS2, ATP5H, NDUFS1, ATP5J, ND1, NDUFB11, NDUFB10, ND4, SLC25A4, ATP4A, SLC25A5, ATP4B, ND3, NDUFC2, GIF, NDUFC1, NDUFA10, ATP6V1D, NDUFA12, SLC26A7, UQCRH, CLIC5, CLIC6, SDCCAG3, SORT1, RANGRF, EIF5A2, UQCRB, MYO5A, NDUFB3, NDUFB5, RAB3C, NDUFB6, NDUFB8, NDUFB9, TIMM10, CYTB, ATP5G1, ATP5G3, SEC14L1, TMED6, KCNE2, SLC39A6,

					NDUFA4, NDUFA5, CPT1B, NDUFA2, NDUFA3, NDUFA8, NDUFA9, NDUFA6, ATP5F1, NDUFA1, SDHA, PHAX, SMDT1, SLC16A7, ATP6V1E1, MCFD2, ATP5A1
GOTERM_BP.	GO:0015992~proton transport	23	3.58	0.0000	ATP5E, ATP5J2, ATP4A, ATP4B, ATP5B, ATP5F1, CYTB, ATP5G1, ATP6V1D, ATP5G3, ATP6V0B, NNT, ATP6V1E1, ATP5C1, ATP5L, SLC9B2, ATP5O, ATP5A1, ATP6V1G3, ATP5H, ATP8, ATP5J, ATP6
GOTERM_BP.	GO:0015986~ATP synthesis coupled proton transport	13	2.02	0.0000	ATP5E, ATP5B, ATP5F1, ATP5G1, ATP5G3, ATP5C1, ATP5L, ATP5O, ATP5A1, ATP5H, ATP8, ATP5J, ATP6
GOTERM_BP.	GO:0006122~mitochondrial electron transport, ubiquinol to cytochrome c	10	1.56	0.0000	UQCRC2, UQCC3, UQCR10, UQCRC1, UQCRH, CYC1, CYCS, CYTB, UQCRQ, UQCRB
GOTERM_BP.	GO:0006754~ATP biosynthetic process	12	1.87	0.0000	ATP5E, UQCC3, ATP5J2, CHCHD10, ATP5B, GBAS, ATP5C1, ATP5L, ATP5O, ATP5A1, ATP8, ATP6
GOTERM_BP.	GO:0006120~mitochondrial electron transport, NADH to ubiquinone	9	1.40	0.0000	NDUFS6, ND4, NDUFB8, NDUFB9, DLD, NDUFV2, NDUFC2, COQ9, NDUFA10
GOTERM_BP.	GO:0006099~tricarboxylic acid cycle	12	1.87	0.0000	SDHA, DLST, SUCLG1, CS, IDH2, IDH3B, DLAT, PDHA1, PDHB, MDH2, IDH3A, MDH1

GOTERM_BP.	GO:0046034~ATP metabolic process	12	1.87	0.0000	ATP5J2, ATP5B, AK1, AK3, ATP5C1, ATP5L, ATP5O, ATP5A1, ATP5G1, ATP5H, NDUFS1, ATP5J
GOTERM_BP.	GO:0006811~ion transport	46	7.15	0.0001	KCNJ16, SLC22A16, ATP5E, SLC9A7, SCN1B, LRRC8B, GABRB1, ATP5B, ATP5G1, ATP5G3, ATP6V0B, SLC30A4, SLC9B2, ATP5L, SLC22A3, KCNE2, ATP5O, SLC4A2, SLC39A6, NALCN, ATP5H, ATP8, ATP6, ATP5J, HCN1, HCN2, ATP5J2, KCND2, ATP4A, ATP4B, ATP5F1, TOMM40, GIF, VDAC2, ATP6V1D, VDAC3, SMDT1, SLC26A7, ATP6V1E1, CLIC5, TOMM40L, CLIC6, ATP5C1, ATP5A1, ATP6V1G3, ATP7B
GOTERM_BP.	GO:0006103~2-oxoglutarate metabolic process	8	1.24	0.0012	GOT2, MRPS36, DLST, GOT1, DLD, IDH2, IDH3B, IDH3A
GOTERM_BP.	GO:0006123~mitochondrial electron transport, cytochrome c to oxygen	7	1.09	0.0013	COX8B, CYCS, COX7C, COX4I1, COX6A1, COX5A, COX5B
GOTERM_BP.	GO:0022904~respiratory electron transport chain	7	1.09	0.0317	SDHA, NDUFA5, NDUFS6, COX3, CYTB, PPARGC1A, SOD2
GOTERM_BP.	GO:1902600~hydrogen ion transmembrane transport	7	1.09	0.0317	UQCR10, COX8B, COX7B, ATP5G1, ATP5G3, ATP6V0B, UQCRB

GOTERM_CC.	GO:0005739~mitochondrion	217	33.75	0.0000	HCCS, LDHB, TMEM143, HIBADH, GOT2, SLC9B2, PDHA1, ATP8, GNG5, TMEM14C, MRPL34, ATP6, MRPL35, APOO, MRPL53, BSG, MRPL51, SUCLG1, GLUL, NNT, CD36, DLD, MRPL49, ATP5C1, ATPIF1, MDH2, MDH1, HSD17B10, ACADSB, TXN2, CHCHD3, CHCHD2, ACAT1, IDH2, PEMT, COX6B1, MRPL55, PHYHIPL, PHB, JTB, MPC2, ACACB, DLAT, VDAC2, AFG3L2, VDAC3, CHCHD10, COX3, COX2, COX1, HEBP2, COX6A1, BCO2, ARSB, SLC27A1, ATP5E, TIMM17A, ATP5B, SLIRP, CKB, ASAH2, GBAS, SLC25A3, ATP5L, ATP5O, ADCY10, ATP5H, COX17, DNAJC19, ATP5J, NDUFB11, NDUFB10, SLC25A4, SLC25A5, AIFM1, NDUFA13, NDUFA10, NDUFA12, PFDN4, BCAT2, AASS, COX7A2L, MRPL11, MMACHC, CKMT2, MRPL16, REXO2, MARS, ECI2, CPT1B, COX7A2, IMMT, CS, SOD1, SOD2, CAPRIN2, SDHA, PRELID2, MRPL21, TFRC, UOX, MRPL27, ATP6V1E1, POLDIP2, BNIP3L, ATP5A1, MRPS36, NDUFAB1, ROMO1, COX5A, COX5B, PDHB, FAHD1, UQCR10, GHITM, UQCR11, CISD3, SLC25A23, TCAIM, HADH, STAP1, TMEM126A, SLC25A30, ISCU, PYCR2, MRPS18B, SLC25A35, ME3, HACD3, COX7B, COX7C, GADD45GIP1, TOMM5, MTCH1, BLOC1S1, HSPE1, ATP5J2, COX8B, AK3, IDH3B, TOMM40, IDH3A, NDUFV3, SLC25A11, USMG5, SLC25A10, TOMM40L, TDH, NDUFV2, MYH10, UQCRC2, ETNPPL, ACOX2, CYB5R1, UQCRC1, SGPP1, HINT2, CYC1, PRDX5, PRDX2, PRDX3, UQCRFS1, UQCRQ, NDUFS7, NDUFS6, NDUFS8, PXMP2, NDUFS3, NDUFS2, NDUFS1, ND1, SUOX, ND4, ND3, NOL7, CYCS, NDUFC2, COX4I1, COQ9, NDUFC1, COQ6, ISCA1, C1QBP, UQCRH, PPM1K, PEBP1, OAT, UQCRB, NDUFB3, NDUFB5, NDUFB6, NDUFB8, NDUFB9, FKBP4, TIMM10, CYTB, ATP5G1, PTPMT1, ATP5G3, SNN, OXCT1, HSPA9, NDUFA4, NDUFA5, DLST, NDUFA2, NDUFA3, NDUFA8, NDUFA9, NDUFA6, ATP5F1, NDUFA1, UQCC3, DBT, SMDT1, BOLA1,

RDH14

GOTERM_CC.	GO:0005743~mitochondrial inner membrane	108	16.80	0.0000	HCCS, ROMO1, COX5A, COX5B, GOT2, FAHD1, UQCR10, UQCR11, GHITM, SLC25A23, HADH, TMEM14C, ATP6, APOO, SUCLG1, TMEM126A, SLC25A30, NNT, SLC25A35, ATP5C1, MDH2, HSD17B10, COX7B, COX7C, CHCHD3, ACAT1, MTCH1, COX6B1, IDH2, ATP5J2, LGALS3, COX8B, PHB, MPC2, AFG3L2, VDAC2, VDAC3, NDUFV3, SLC25A11, USMG5, COX3, COX2, COX1, SLC25A10, NDUFV2, COX6A1, UQCRC2, ATP5E, SLC27A1, UQCRC1, ATP5B, TIMM17A, CYC1, UQCRFS1, UQCRQ, NDUFS7, NDUFS6, SLC25A3, ATP5L, ATP5O, NDUFS3, ATP5H, NDUFS2, DNAJC19, NDUFS1, ATP5J, ND1, NDUFB11, NDUFB10, SLC25A4, AIFM1, SLC25A5, NDUFC2, COX4I1, NDUFA13, COQ9, NDUFC1, NDUFA12, COQ6, UQCRH, UQCRB, NDUFB3, NDUFB5, NDUFB6, NDUFB8, NDUFB9, TIMM10, CYTB, ATP5G1, COX7A2L, PTPMT1, CKMT2, NDUFA4, NDUFA5, NDUFA2, NDUFA3, COX7A2, NDUFA8, NDUFA9, IMMT, NDUFA6, ATP5F1, NDUFA1, SOD2, SDHA, UQCC3, SMDT1, ATP5A1
GOTERM_CC.	GO:0070469~respiratory chain	44	6.84	0.0000	UQCRC2, NDUFB3, NDUFB5, NDUFB6, UQCRC1, NDUFB8, NDUFB9, CYC1, NDUFAB1, CYTB, UQCRFS1, UQCRQ, NDUFS7, NDUFS6, UQCR10, UQCR11, NDUFS8, NDUFS3, NDUFS2, NDUFS1, NDUFA4, ND1, NDUFA5, NDUFA2, NDUFB11, NDUFA3, NDUFB10, ND4, NDUFA8, NDUFA9, NDUFA6, ND3, CYCS, NDUFC2, NDUFC1, NDUFA10, NDUFA1, NDUFA12, NDUFV3, COX2, UQCRH, COX1, NDUFV2, UQCRB
GOTERM_CC.	GO:0005747~mitochondrial respiratory chain complex I	32	4.98	0.0000	NDUFB3, NDUFB5, NDUFB6, NDUFB8, NDUFB9, NDUFAB1, NDUFS7, NDUFS6, NDUFS8, NDUFS3, NDUFS2, NDUFS1, NDUFA4, NDUFA5, ND1, NDUFB11, NDUFA2, NDUFA3, NDUFB10, NDUFA8, ND4, NDUFA9, NDUFA6, ND3, NDUFC2, NDUFA13, NDUFC1, NDUFA10, NDUFA1,

					NDUFA12, NDUFV3, NDUFV2
GOTERM_CC.	GO:0043209~myelin sheath	46	7.15	0.0000	UQCRC2, LDHB, UQCRC1, ATP5B, PGAM1, RDX, PRDX2, CCT2, PRDX3, COX5A, UQCRFS1, COX5B, CKB, GOT2, SLC25A3, ATP5O, MSN, PDHA1, PLCB1, NDUFS3, ATP5H, NDUFS1, HSPA9, DLST, SLC25A4, IMMT, SLC25A5, PHB, CYCS, ATP5F1, DLAT, NDUFA10, VDAC2, SOD1, IDH3A, SOD2, SDHA, GLUL, DLD, NDUFV2, ATP5C1, COX6A1, PEBP1, ATP5A1, MDH2, MDH1
GOTERM_CC.	GO:0005753~mitochondrial proton-transporting ATP synthase complex	15	2.33	0.0000	ATP5E, ATP5J2, ATP5B, ATP5F1, ATP5G1, USMG5, ATP5C1, ATP5L, ATP5O, ATPIF1, ATP5A1, ATP5H, ATP8, ATP5J, ATP6
GOTERM_CC.	GO:0016020~membrane	323	50.23	0.0000	HCCS, LDHB, SLC9A7, CADM4, SGMS2, PGAM1, AQP4, TMEM143, SIDT2, KLHDC7A, GOT2, HTR1B, TMEM145, DIRAS2, GAB2, ILK, VMA21, SLC9B2, MUC15, ATP8, GNG5, TMEM14C, CDH24, ATP6, APOO, C5AR2, BSG, MDGA1, HTR4, LIFR, PLAUR, RND2, CD36, NNT, UNC13D, CCR5, SSTR1, ATP5C1, RAB12, ATP6V1G3, MDH2, SUCO, SCN1B, HSD17B12, ENPP4, CHCHD3, TMCC3, ATP6V0B, CDC42EP1, DMD, PEMT, PABPC1, GCNT1, LAMTOR1, LGALS3, LAMTOR2, PHB, PMEL, JTB, MPC2, ACACB, VDAC2, AFG3L2, VDAC3, KDR, EPHA5, CLGN, EPHA6, COX3, COX2, COX1, COX6A1, GRK1, NXPE2, KCNJ16, SLC27A1, ATP5E, SLC16A10, TIMM17A, ATP5B, TMEM161B, RTN1, INSRR, ASAH2, VEPH1, POMGNT1, MAP1LC3A, SLMAP, SERPINA5, SLC25A3, SLC22A3, ATP5L, ATP5O, ADCY10, ATP5H, DPEP1, USH2A, ATP5J, ZDHHC2, NDUFB11, NDUFB10, GPR176, PIK3C2G, SLC25A4, ATP4A, AIFM1, SLC25A5, ATP4B, NDUFA12, ZDHHC14, IL20RA, SLC26A7, CLIC5, CLIC6, PTGDR, SORT1, VOPP1, STBD1, EIF5A2, MYO5A, RAB3C, RPL36, COX7A2L, KIT, FNDC4, PCNX2, CKMT2, KCNE2, C1GALT1,

		DTNA, MARS, ECI2, CPT1B, A4GALT, COX7A2, IMMT, NPR3, APPL1, FZD7, SDHA, TFRC, SLC16A7, CDH16, CDH18, BNIP3L, MBOAT1, MYCBPAP, MBOAT2, MBOAT4, ATP5A1, PXYLP1, PGK1, PHLDB2, SLC22A16, GABRB1, SLC7A8, ROMO1, COX5A, SLC7A6, ST3GAL1, UQCR10, DYSF, UQCR11, GHITM, SLC2A4, TIAM2, ST3GAL4, ANK3, SLC25A23, ELOVL3, ST3GAL6, SLC4A2, PDGFD, NALCN, PRKACB, RNF149, RNF34, MATK, CUTA, TMEM203, KCND2, SMIM13, TMEM126A, SLC25A30, ABHD17B, SEC61B, SLC25A35, DYNLRB1, FILIP1L, SNX9, GAL3ST3, ARHGDIG, HACD3, COX7B, NKAIN2, COX7C, PAQR3, IKBIP, GPR22, TOMM5, APOBR, BLOC1S1, MTCH1, TMEM30C, SLC30A4, SLC35F3, HSPE1, SDF2, HCN1, HCN2, ATP5J2, CCPG1, COX8B, TMEM92, TOMM40, SNAPIN, WIPI1, ABCB5, NDUFV3, SLC25A11, USMG5, ATF4, CRBN, GPR34, PPIB, SLC25A10, PLCG2, TOMM40L, NDUFV2, SPCS1, KCTD16, ATP7B, UQCRC2, CYB5R1, UQCRC1, SGPP1, LRRC8B, PRKAG1, CYC1, UQCRFS1, UQCRQ, NDUFS6, PRMT1, SLC1A2, PLEKHB1, TMEM171, HMOX1, NDUFS8, PXMP2, PIGC, LOXL4, MSN, PLCB1, NDUFS3, NDUFS2, IMPDH2, NDUFS1, ND1, BTNL9, PTGER3, ND4, ND3, TMEM255B, NDUFC2, COX4I1, NDUFC1, KRTCAP2, ATP6V1D, LDLRAD4, COQ6, LYPD6B, LARP4, C1QBP, UQCRH, CHST9, EMC6, NKIRAS1, UQCRB, FKBP2, NDUFB3, CYP2U1, NDUFB5, NDUFB6, FKBP5, NDUFB8, NDUFB9, TIMM10, CYTB, TMEM35B, RDX, PTPMT1, ATP5G1, ATP5G3, TMEM258, TMED6, RGMA, SNN, COL17A1, RASGRP1, SYN3, RASGRP2, MYZAP, RNF128, PALM3, SLC39A6, TMEM45A, NDUFA4, DLST, NDUFA5, NDUFA2, NDUFA3, NDUFA8, FLT3, NDUFA6, ATP5F1, IGSF9B, NDUFA1, TMEM55A, UQCC3, TMEM47, SMDT1, BMPR1B, RDH14

GOTERM_CC.	GO:0070062~extracellular exosome	152	23.64	0.0000	SRP14, GNA14, LDHB, CADM4, FAM20A, RUSC2, FAM20C, PGAM1, SLC7A8, CCT2, COX5A, SIDT2, PDHB, ST3GAL1, GOT2, UQCR10, DYSF, GHITM, GOT1, SLC2A4, TIAM2, ST3GAL4, ST3GAL6, ROBO4, IGLL1, RNF149, PDGFD, PRKACB, GNG5, ATP6, CUTA, BSG, PDXK, TNIK, SUCLG1, LIFR, PDXP, PLAUR, GLUL, ATP5C1, MDH2, MDH1, SNX9, BPIFB1, ACADSB, ENPP4, CHCHD3, PIN4, ACAT1, TPI1, PSMB6, FGA, BLOC1S1, CACYBP, IDH2, ENTPD6, EIF3I, HSPE1, PABPC1, GPR155, ATP5J2, ACY1, LAMTOR1, LGALS3, LAMTOR2, AK1, PHB, TOMM40, PTPN13, VDAC2, SERPINI1, VDAC3, EMILIN1, USMG5, DNAJB9, PPIB, COX2, HEBP2, PLCG2, FABP3, MYH10, UQCRC2, ARSB, CYB5R1, OPCML, PRKAG1, HINT1, ATP5B, ESD, RASSF9, PRDX5, PRDX2, PRDX3, YBX1, CKB, SERPINA5, ACOT11, SLC25A3, ITIH4, ATP5L, ATP5O, LOXL4, MSN, PLCB1, ATP5H, IMPDH2, DPEP1, ZCCHC11, NDUFB10, SLC25A5, NDUFA13, COX4I1, ATP6V1D, CTH, CLIC5, DDT, CLIC6, UBE2M, PEBP1, CTSF, FKBP2, MYO5A, NDUFB3, FKBP5, FKBP4, NDUFB9, RDX, RGMA, ANXA9, MARS, HSPA9, NDUFA4, DLST, GSTA3, LYZ2, A4GALT, COX7A2, LYZ1, PM20D2, CS, ATP5F1, NPR3, KLK1, APPL1, SOD1, SOD2, TFRC, CDH16, ATP6V1E1, ATP5A1, PGK1, PRSS23
GOTERM_CC.	GO:0005750~mitochondrial respiratory chain complex III	9	1.40	0.0000	UQCRC2, UQCC3, UQCR10, UQCRC1, UQCRH, CYC1, UQCRFS1, UQCRQ, UQCRB
GOTERM_CC.	GO:0045263~proton-transporting ATP synthase complex, coupling factor F(o)	9	1.40	0.0000	ATP5J2, ATP5F1, ATP5L, ATP5G1, ATP5H, ATP5G3, ATP8, ATP6, ATP5J
GOTERM_CC.	GO:0000276~mitochondrial proton-transporting ATP synthase complex, coupling factor F(o)	9	1.40	0.0000	ATP5J2, ATP5F1, ATP5L, ATP5O, ATP5G1, ATP5H, ATP5G3, ATP8, ATP5J
GOTERM_CC.	GO:0005751~mitochondrial respiratory chain complex IV	9	1.40	0.0000	NDUFA4, COX8B, COX2, COX1, COX7C, COX4I1, COX6A1, COX5A, COX5B

GOTERM_CC.	GO:0005759~mitochondrial matrix	23	3.58	0.0002	ME3, ACADSB, NDUFA9, SUCLG1, CS, AK3, NDUFA10, ACAT1, PDHB, GOT2, ISCA1, ISCU, C1QBP, PPM1K, OXCT1, BLOC1S1, REXO2, DLD, HSPE1, PDHA1, HADH, OAT, MDH2
GOTERM_CC.	GO:0005758~mitochondrial intermembrane space	14	2.18	0.0007	SUOX, PRELID2, CHCHD10, NDUFA8, AIFM1, BLOC1S1, REXO2, CYCS, COX6B1, AK3, CHCHD2, SOD1, COX17, NDUFS1
GOTERM_CC.	GO:0031966~mitochondrial membrane	14	2.18	0.0223	NDUFA2, NDUFB6, NDUFA9, ATP5B, NDUFA6, ND3, COX4I1, NDUFA13, UQCRFS1, NDUFA1, SLC25A10, PEMT, SLC9B2, NDUFS3
