# Supplementary material for: The Effects of Artificially Dosed Adult Rumen Contents on Abomasum Transcriptome and Associated Microbial Community Structure in Calves
Source: Genes (Basel). 2021 Mar 16;12(3):424. doi: 10.3390/genes12030424 (PMC7999174; doi:10.3390/genes12030424)
Supplement: Supplementary file 1 [file genes-12-00424-s001.zip › Supplementary Table 3-genera.pdf]

The effects of artificially dosed adult rumen contents on abomasum transcriptome and associated microbial community structure in calves

Naren Gaowa[1], Wenli Li[2]*, Brianna Murphy[2], Madison Cox[3]

1 State Key Laboratory of Animal Nutrition, Beijing Engineering Technology, Research Center of Raw Milk Quality and Safety Control, College of Animal Science and Technology, China Agricultural University, No.2 Yuanmingyuan West Road, Haidian, Beijing 100193, China.

2 The Cell Wall Utilization and Biology Laboratory, USDA Agricultural Research Service, US Dairy Forage Research Center, Madison, WI 53706, USA

3 Department of Microbiology, University of Wisconsin-Madison, Madison, WI, 53706, USA

*Correspondence: wenli.li@ars.usda.gov

Supplementary Table 3 Genera in the abomasum between HE and Con groups

Genus	Con	HE	Pvalue
Desulfovibrio	185.17	337.16	0.0209
Leptotrichia	11.16	3.05	0.0421
Tannerella	61.72	512.06	0.0433
Eubacterium	517.69	154.89	0.0433
Deinococcus	4.07	0.00	0.0472
Dehalococcoides	50.59	23.32	0.0833
Methylobacterium	5.34	33.21	0.0833
Streptomyces	1.63	0.00	0.1306
Rickettsia	4.77	2.61	0.1391
Helicobacter	12.61	5.19	0.1465
Clostridium	3540.71	9810.74	0.1489
Acinetobacter	3004.76	1729.05	0.1489
Hymenobacter	46.93	152.26	0.1489
Francisella	422.09	225.16	0.1489
Ruminiclostridium	176.41	63.17	0.1489
Capnocytophaga	129.22	87.64	0.1489
Hyphomicrobium	31.87	16.89	0.1489
Thermotoga	4.14	0.69	0.1663
Spirosoma	128.08	5.07	0.2367
Algoriphagus	41.32	39.16	0.2367
Chlamydia	0.89	6.97	0.2367
Kocuria	10.00	10.80	0.2454
Nitrosospira	3604.57	1956.52	0.2482
Sphaerochaeta	382.07	312.80	0.2482
Staphylococcus	252.72	495.23	0.2482
Mycoplasma	165.52	322.03	0.2482
Halanaerobium	21.17	43.15	0.2482
Thermoanaerobacterium	171.69	20.03	0.2482
Exiguobacterium	179.60	38.39	0.2482
Bartonella	8.25	665.97	0.2482
Synechococcus	107.39	82.34	0.2482
Ochrobactrum	181.69	115.53	0.2482
Pseudomonas	95.28	65.29	0.2482
Flavobacterium	10.92	117.24	0.2482
Kangiella	16.83	38.61	0.2482
Entomoplasma	0.98	0.00	0.3173
Rubrobacter	0.24	0.00	0.3173
Fervidobacterium	0.24	0.00	0.3173
Ruminococcus	2109.88	3084.00	0.3865
Parabacteroides	2190.15	2244.44	0.3865
Alistipes	155.96	244.54	0.3865

Selenomonas	2436.65	1665.58	0.3865
Desulfosporosinus	46.56	33.00	0.3865
Dickeya	326.98	236.35	0.3865
Olsenella	92.99	211.50	0.3865
Salmonella	103.57	208.10	0.3865
Methanobrevibacter	73.53	56.94	0.3865
Blattabacterium	19.34	29.41	0.3865
Comamonas	5.25	37.01	0.3865
Calothrix	1.93	14.53	0.4419
Candidatus	3.52	25.28	0.4678
Burkholderia	0.24	2.84	0.5083
Alkaliphilus	0.95	19.75	0.5385
Sphingomonas	5.78	4.34	0.5541
Fusobacterium	15.63	13.25	0.5614
Prevotella	50205.65	44085.48	0.5637
Lachnoclostridium	3311.75	4002.15	0.5637
Butyrivibrio	5634.20	6216.90	0.5637
Treponema	2904.58	2257.47	0.5637
Lactobacillus	1911.82	2487.17	0.5637
Anaerococcus	460.78	284.04	0.5637
Tumebacillus	47.19	120.04	0.5637
Psychrobacter	399.19	273.57	0.5637
Campylobacter	911.47	760.14	0.5637
Bifidobacterium	214.24	1435.58	0.5637
Desulfotomaculum	118.87	125.68	0.5637
Neisseria	690.59	331.39	0.5637
Veillonella	317.46	280.41	0.5637
Dehalobacter	172.07	116.81	0.5637
Aeromonas	311.70	488.57	0.5637
Paenibacillus	231.76	155.67	0.5637
Listeria	10.20	55.44	0.5637
Bdellovibrio	109.27	66.05	0.5637
Streptococcus	53.22	265.80	0.5637
Aerococcus	60.35	38.02	0.5637
Leuconostoc	129.70	44.69	0.5637
Brevibacillus	28.32	52.59	0.5637
Corynebacterium	27.59	61.14	0.5637
Geobacter	21.38	22.98	0.5637
Pseudoalteromonas	5.84	36.72	0.5637
Taylorella	33.66	17.54	0.5637
Porphyromonas	6.75	7.45	0.7674
Rhodococcus	7.76	27.66	0.7715
Moraxella	209.41	149.58	0.7728

Acholeplasma	43.20	67.02	0.7728
Ureaplasma	129.37	179.92	0.7728
Rufibacter	74.18	112.85	0.7728
Nitrosomonas	139.24	44.17	0.7728
Erysipelothrix	32.27	38.67	0.7728
Spiroplasma	26.39	37.16	0.7728
Geobacillus	8.04	25.39	0.8845
Planococcus	20.22	18.47	0.8845
Chryseobacterium	4.54	11.58	0.8845
Bacteroides	4523.94	4295.69	1.0000
Blautia	4185.05	3934.08	1.0000
Acidaminococcus	517.48	609.05	1.0000
Mucilaginibacter	193.05	348.34	1.0000
Pelosinus	255.77	255.21	1.0000
Bacillus	93.80	93.31	1.0000
Desulfitobacterium	8.98	18.86	1.0000
Enterococcus	13.37	13.81	1.0000
Vibrio	5.10	6.62	1.0000
