# Supplementary material for: The Effects of Artificially Dosed Adult Rumen Contents on Abomasum Transcriptome and Associated Microbial Community Structure in Calves
Source: Genes (Basel). 2021 Mar 16;12(3):424. doi: 10.3390/genes12030424 (PMC7999174; doi:10.3390/genes12030424)
Supplement: Supplementary file 1 [file genes-12-00424-s001.zip › Supplementary Table S4.doc]

**Table S4:** The Pearson r-value and p-value between the genera and the genes enriched in the immune response.

| **Terms** | ***Tannerella*** | | ***Desulfovibrio*** | | ***Deinococcus*** | | ***Leptotrichia*** | | ***Eubacterium*** | |
| --- | --- | --- | --- | --- | --- | --- | --- | --- | --- | --- |
|  | **r-value** | **p-value** | **r-value** | **p-value** | **r-value** | **p-value** | **r-value** | **p-value** | **r-value** | **p-value** |
| *MX1* | -0.1 | 0.87 | 0.3 | 0.47 | -0.4 | 0.36 | -0.3 | 0.51 | -0.4 | 0.37 |
| *HERC6* | -0.1 | 0.84 | 0.2 | 0.61 | -0.3 | 0.40 | -0.2 | 0.56 | -0.3 | 0.48 |
| *LGALS9* | 0.3 | 0.43 | 0.5 | 0.18 | -0.5 | 0.22 | -0.5 | 0.18 | -0.4 | 0.28 |
| *RSAD2* | -0.1 | 0.87 | 0.4 | 0.38 | -0.4 | 0.30 | -0.2 | 0.61 | -0.4 | 0.37 |
| *ZBP1* | -0.1 | 0.90 | 0.2 | 0.67 | -0.4 | 0.32 | -0.3 | 0.41 | -0.3 | 0.43 |
| *ADAR* | -0.1 | 0.80 | 0.3 | 0.52 | -0.4 | 0.30 | -0.4 | 0.38 | -0.4 | 0.29 |
| *IFIH1* | 0 | 0.94 | 0.3 | 0.48 | -0.4 | 0.36 | -0.3 | 0.54 | -0.3 | 0.53 |
| *DDX58* | 0 | 0.94 | 0.3 | 0.54 | -0.4 | 0.39 | -0.4 | 0.37 | -0.4 | 0.30 |
| *TRIM25* | 0 | 0.99 | 0.4 | 0.30 | -0.4 | 0.31 | -0.3 | 0.41 | -0.4 | 0.36 |
| *SRC* | 0.1 | 0.80 | 0.5 | 0.23 | -0.5 | 0.23 | -0.2 | 0.61 | -0.2 | 0.56 |
| *MST1R* | 0.1 | 0.90 | 0.4 | 0.33 | -0.4 | 0.39 | -0.3 | 0.42 | -0.3 | 0.43 |
| *DHX58* | -0.1 | 0.79 | 0.5 | 0.23 | -0.4 | 0.30 | -0.2 | 0.69 | -0.4 | 0.34 |
| *ISG20* | 0 | 0.95 | 0.3 | 0.46 | -0.5 | 0.18 | -0.3 | 0.40 | -0.4 | 0.33 |
| *PML* | 0.1 | 0.84 | 0.4 | 0.37 | -0.2 | 0.58 | -0.5 | 0.22 | -0.4 | 0.28 |
| *IFIT3* | -0.1 | 0.87 | 0.3 | 0.41 | -0.4 | 0.31 | -0.2 | 0.55 | -0.4 | 0.38 |
| *OAS2* | -0.1 | 0.81 | 0.3 | 0.41 | -0.4 | 0.32 | -0.3 | 0.41 | -0.5 | 0.22 |
| *LY86* | 0.5 | 0.22 | 0.4 | 0.31 | -0.5 | 0.25 | -0.3 | 0.46 | -0.2 | 0.63 |
| *IRF5* | 0.2 | 0.68 | 0.5 | 0.16 | -0.5 | 0.16 | 0 | 0.94 | -0.1 | 0.76 |
| *KCNN4* | 0.1 | 0.85 | 0.5 | 0.16 | -0.6 | 0.10 | 0.1 | 0.85 | -0.1 | 0.78 |
| *PYCARD* | 0.5 | 0.21 | 0.7 | 0.08 | -0.4 | 0.29 | -0.3 | 0.44 | -0.2 | 0.63 |
| *SYK* | 0.3 | 0.44 | 0.6 | 0.15 | -0.5 | 0.25 | -0.2 | 0.71 | -0.1 | 0.76 |
| *TRPM4* | 0.3 | 0.43 | 0.8 | 0.03 | -0.8 | 0.03 | -0.1 | 0.84 | -0.3 | 0.41 |
| *TLR2* | 0.4 | 0.29 | 0.7 | 0.07 | -0.8 | 0.02 | -0.5 | 0.24 | -0.6 | 0.12 |
| *NLRX1* | 0.5 | 0.20 | 0.4 | 0.28 | -0.7 | 0.04 | -0.7 | 0.04 | -0.7 | 0.07 |
